# An integrative transcriptome analysis indicates regulatory mRNA-miRNA networks for residual feed intake in Nelore cattle

**DOI:** 10.1038/s41598-018-35315-5

**Published:** 2018-11-20

**Authors:** Priscila S. N. De Oliveira, Luiz L. Coutinho, Polyana C. Tizioto, Aline S. M. Cesar, Gabriella B. de Oliveira, Wellison J. da S. Diniz, Andressa O. De Lima, James M. Reecy, Gerson B. Mourão, Adhemar Zerlotini, Luciana C. A. Regitano

**Affiliations:** 1 Embrapa Pecuária Sudeste, São Carlos, SP 13560-970 Brazil; 2 Department of Animal Science, University of São Paulo, Piracicaba, SP 13418-900 Brazil; 3 NGS Genomic Solutions, Piracicaba, SP 13418-900 Brazil; 4 Department of Genetics and Evolution, Federal University of São Carlos, São Carlos, SP 13565-905 Brazil; 5 Department of Animal Science, Iowa State University, Ames, IA 50011 USA; 6 Embrapa Informática Agropecuária, Campinas, SP 770901 Brazil

## Abstract

Residual Feed Intake (RFI) is an economically relevant trait in beef cattle. Among the molecular regulatory mechanisms, microRNAs (miRNAs) are an important dimension in post-transcriptional regulation and have been associated with different biological pathways. Here, we performed differential miRNAs expression and weighted gene co-expression network analyses (WGCNA) to better understand the complex interactions between miRNAs and mRNAs expressed in bovine skeletal muscle and liver. MiRNA and mRNA expression data were obtained from Nelore steers that were genetically divergent for RFI (N = 10 [low RFI or feed efficient]; N = 10 [high RFI or feed inefficient]). Differentially expressed and hub miRNAs such as bta-miR-486, bta-miR-7, bta-miR15a, bta-miR-21, bta-miR 29, bta- miR-30b, bta-miR-106b, bta-miR-199a-3p, bta-miR-204, and bta-miR 296 may have a potential role in variation of RFI. Functional enrichment analysis of differentially expressed (DE) miRNA’s target genes and miRNA–mRNA correlated modules revealed that insulin, lipid, immune system, oxidative stress and muscle development signaling pathways might potentially be involved in RFI in this population. Our study identified DE miRNAs, miRNA - mRNA regulatory networks and hub miRNAs related to RFI. These findings suggest a possible role of miRNAs in regulation of RFI, providing new insights into the potential molecular mechanisms that control feed efficiency in Nelore cattle.

## Introduction

Feed efficiency (FE) and residual feed intake (RFI) have been explored as a mean to improve the efficiency of beef production^1^. Feed efficient animals produce fewer pollutants e.g., methane and consume less feed, thereby reducing the cost of beef production, making feed efficiency an economically relevant trait.

Recent functional genomics studies have identified roles for microRNAs (miRNAs) in the regulation of feed efficiency and related-traits^2,3^. MiRNAs are small non-coding RNAs that are highly conserved between species^4^ and are associated with many important biological processes. Among the regulatory mechanisms, miRNAs have emerged as a new dimension in post-transcriptional regulation in mammals, usually suppressing the translation of their target mRNAs by base‐pairing to the 3′ untranslated region (UTR)^5^.

In domestic animals, miRNAs have been reported to be key regulators of development of skeletal muscle, fat and mammary tissues, immune response and fertility^2,6,7^. Expression-profiling studies of miRNAs in domestic livestock have revealed their tissue-specific and temporal expression pattern, with associations with economic traits, highlighting the potential use of miRNAs in future genomic selection programs^8^. Differences in beef cattle miRNA expression patterns have been identified in animals with divergent RFI, which indicates a potential regulatory role of these molecules on this phenotype^3^.

Previous studies using the same Nelore population have identified markers^9^ and differentially expressed genes related to RFI in skeletal muscle^10^ and liver^11^. Biological processes such as immune response, lipid and fatty acid metabolism, energy and growth, xenobiotics metabolism, and oxidative stress were highlighted as significant pathways enriched for this population. However, the variation in feed efficiency involves many biological processes and the miRNA - mRNA regulatory mechanisms that underlie this phenotype are still not clearly understood.

An integrative analysis of miRNA– mRNA expression data might help to unravel the molecular basis and the complex biology of feed efficiency^12^. Weighted Gene Co-expression Network Analysis (WGCNA) has been successfully applied to identify candidate gene networks and hub miRNAs involved with many health disorders^13,14^ and production traits^15,16^. However, miRNA – mRNA co-expression networks related to feed efficiency in cattle have not been evaluated. This study provides a better understanding of the regulatory mechanisms involved in the variation of RFI. Differentially expressed miRNAs, co-expression networks and hub miRNAs from skeletal muscle and liver of animals genetically divergent for RFI were identified. These miRNAs and the co-expressed networks provided evidences of a role in the regulation of genes and biological pathways associated with RFI in this population of Nelore cattle.

## Results

### Phenotypic and miRNA expression data

Best linear unbiased predictions (BLUP; kg/day), phenotypic data for RFI (kg/day), intramuscular fat (IMF; %) and ribeye area (REA; cm^2^) from Nelore steers genetically divergent for RFI are shown in Table 1. A student’s t-test was applied to evaluate the mean differences of BLUP, RFI, IMF and REA between the divergent RFI groups and no significant differences were observed for IMF and REA. It is important to notice that this test was performed on a relatively small sample of animals, which may have influenced the results.

**Table 1:** Best linear unbiased predictions of additive genetic merit (BLUP) for residual feed intake (RFI, kg/day); phenotypic data for RFI (kg/day), intramuscular fat (%) and ribeye area (cm^2^); and the number of miRNA reads mapped per sample for skeletal muscle and liver for feed efficient and feed inefficient RFI groups.

RFI groups	BLUP (Kg/day)	RFI (Kg/day)	IMF (%)	REA (cm^2^)	Mapped reads skeletal muscle	Mapped reads liver
Feed efficient1	−0.0914	−1.0493	2.44	58.75	687,683	410,224
Feed efficient2	−0.0699	−0.5469	3.03	69.75	831,137	749,752
Feed efficient3	−0.0360	−0.5714	2.65	63.00	656,226	475,197
Feed efficient4	−0.0990	−1.2284	3.07	59.00	793,498	620,751
Feed efficient5	−0.0862	−0.7682	3.05	57.25	763,883	470,995
Feed efficient6	−0.0414	−0.6588	3.37	57.00	626,009	614,771
Feed efficient7	−0.0341	−0.3803	2.71	61.25	899,321	524,653
Feed efficient8	−0.0417	−0.1459	2.38	58.00	1,234,435	355,181
Feed efficient9	−0.0679	−1.1983	—	69.00	696,224	541,411
Feed efficient10	−0.0306	−0.2845	4.58	67.75	647, 676	845,567
**Mean**	−**0**.**0598**^**a**^	−**0**.**6832**^**a**^	**3**.**03**^**a**^	**60**.**16**^**a**^	**798**,**713**	**560**,**850**
Feed inefficient1	0.0856	0.3270	3.86	52.75	572,668	464,497
Feed inefficient2	0.0939	0.6588	—	55.75	738,148	663,884
Feed inefficient3	0.0876	0.4115	3.40	56.0	743,922	227,735
Feed inefficient4	0.0480	0.2443	2.34	59.50	654,784	458,327
Feed inefficient5	0.0721	−0.1548	3.69	59.50	1,046,274	440,254
Feed inefficient6	0.1247	1.8084	4.20	66.75	650,387	562,264
Feed inefficient7	0.0875	0.4206	2.86	57.25	804,344	578,310
Feed inefficient8	0.0688	−0.2976	2.83	63.50	842,640	494,721
Feed inefficient9	0.0861	1.2807	3.26	45.75	983,683	585,950
Feed inefficient10	0.0924	0.5969	2.59	53.75	1,263,736	496,194
**Mean**	**0**.**0847**^**b**^	**0**.**5296**^**b**^	**3**.**26**^**a**^	**56**.**63**^**a**^	**830**,**059**	**497**,**214**

Feed efficient 1, 2, 3, 4, 5, 6, 7, 8, 9, 10: Nelore animal IDs; Feed inefficient 1, 2, 3, 4, 5, 6, 7, 8, 9, 10: Nelore animals IDs; a, b: mean differences evaluated by a student’s test.

MiRNA sequencing of small RNA libraries derived from skeletal muscle (N = 10 Feed efficient, N = 10 Feed inefficient) and liver (N = 10 Feed efficient, N = 10 Feed inefficient) yielded 1,359,563 and 1,327,272 million sequences, respectively (Table 1), that ranged from 20–25 bp. On average, 85% and 75% of reads for skeletal muscle and liver samples, respectively, were mapped to *Bos taurus* UMD 3.1 (Ensembl 84: Mar 2016) the genome assembly. In total, 426 skeletal muscle and 342 liver mature miRNAs were detected by miRDeep2 software (Supplementary Table S1), which were considered in the differential expression analysis.

### Differentially expressed miRNAs for feed efficiency and target genes identification

We identified 156 and 154 unique mature miRNA sequences with nonzero expression, for skeletal muscle and liver samples (Supplementary Table S2). One miRNA; bta-miR-486, in skeletal muscle; and four miRNAs; bta-miR-423-5p, bta-miR-30b-5p, bta-miR-339, bta-miR-378, in liver, were differentially expressed (DE) in animals with extreme values for RFI (Table 2). Negative fold-change values indicate lower miRNA expression, thus, all DE miRNAs were down-regulated in feed efficient animals. The target gene list from each DE miRNA from skeletal muscle and liver were predicted with TargetScan and miRanda software. After this approach, the target gene list was filtered by skeletal muscle^10^ and liver^11^ mRNA expression data previously analysed on the same set of samples.

**Table 2:** Differentially expressed miRNAs identified by miRDeep2 between feed efficient and feed inefficient Nelore steers with divergent residual feed intake (RFI) groups and predicted target genes for each miRNA.

miRNA	log2 Fold Change^a^	FDR^b^	Feed efficient^c^	Feed inefficient^d^	Predicted target genes^e^
**Skeletal muscle**					
bta-miR-486	−0.89	0.0578	76,299	105,09	1,360
**Liver**					
bta-miR-423-5p	−1.00	0.0002	270,65	483,10	1,749
bta-miR-30b-5p	−0.67	0.0143	211,02	356,57	784
bta-miR-339a/b	−0.63	0.0143	239,62	345,80	1,486
bta-miR-378	−0.58	0.0171	144,68	249,30	1,331

^a^Log2 Fold Change of Feed efficient to Feed inefficient groups.

^b^False discovery rate adjusted p-values by Benjamini-Hochberg (1995) methodology.

^c,d^Normalized mean counts of Feed efficient and Feed inefficient groups.

^e^Number of predicted target genes.

### Functional enrichment of potential target genes

The Over-representation Enrichment Analysis (ORA) using the target genes list of the DE miRNAs (Supplementary Table S3) performed by WebGestalt software (WEB-based Gene SeT AnaLysis Toolkit^17^), identified significant (FDR < 0.10) and key signaling pathways related to RFI (Table 3).

**Table 3:** Top signaling pathways identified by WebGestalt software for the differentially expressed miRNAs in Nelore steers with divergent residual feed intake (RFI).

miRNA	Signaling Pathways	FDR^a^	Target genes^b^
**Skeletal muscle**			
bta-miR-486	Insulin signaling pathway	0.0566	19
**Liver**			
bta-miR-423-5p	Rap1 signaling pathway	0.0827	32
bta-miR-30b-5p	FoxO signaling pathway	0.0456	13
bta-miR-339a/b	FoxO signaling pathway	0.0001	25
	Insulin signaling pathway	0.0002	24
bta-miR-378	Insulin signaling pathway	0.0704	18

^a^False discovery rate (FDR) adjusted p-values by Benjamini-Hochberg (1995) methodology.

^b^Number of target genes for each specific pathway.

**Figure 1:**
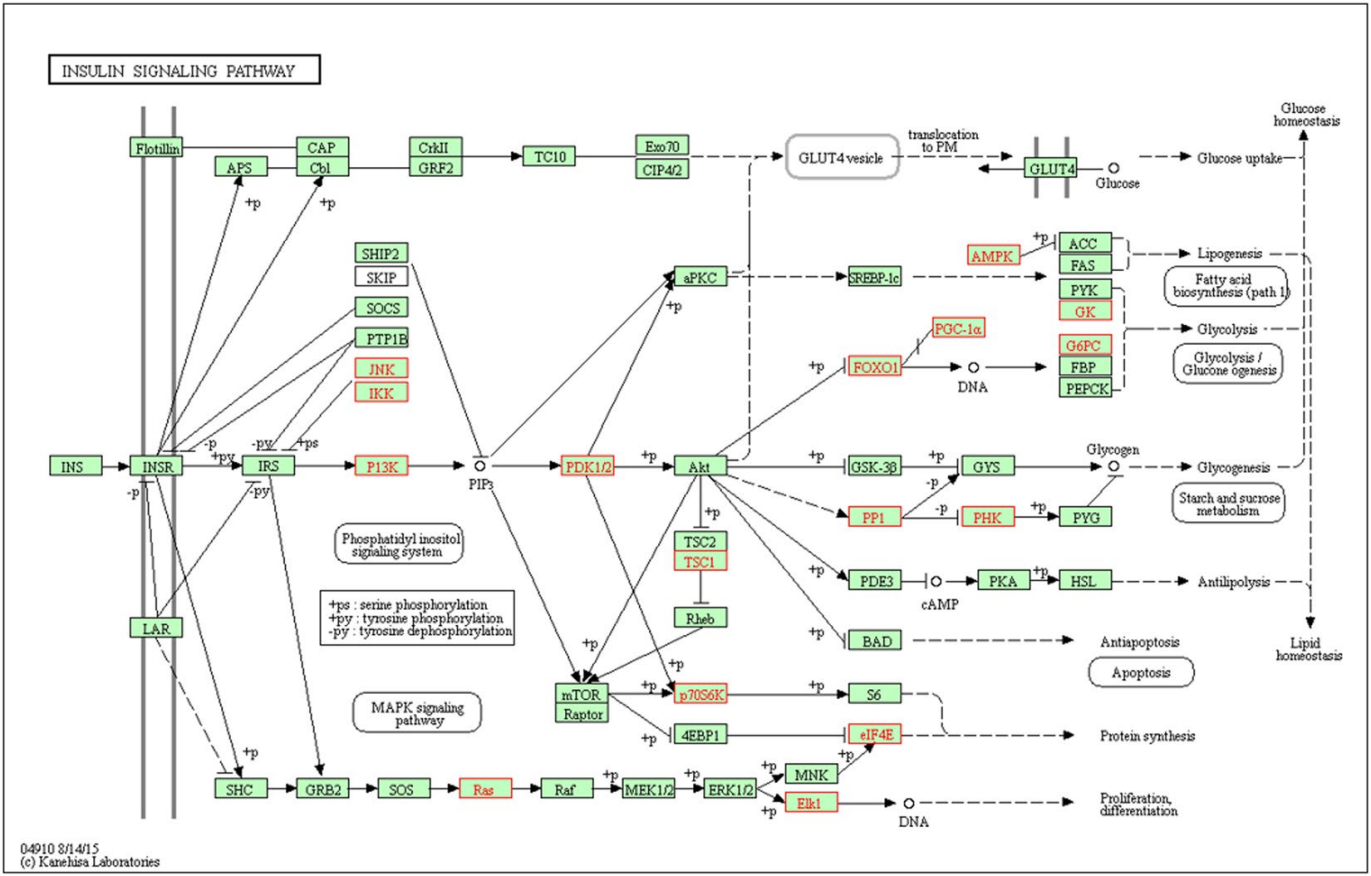
KEGG^56^ insulin signaling pathway identified by WebGestalt from the bta-miR-486 target gene list. Boxes with red labeling indicates target genes for the DE miRNA, while boxes with black labeling are not targeted genes. Solid lines mean direct interaction and dashed lines an indirect interaction between genes.

The insulin signaling pathway was over-enriched based on the target genes of the DE miRNAs bta-miR-486 (skeletal muscle), bta-miR-339a/b and bta-miR-378 (liver). *PPARGC1A* (PPARG coactivator 1 alpha, represented as *PGC-1α*) and *AMPK* (protein kinase AMP-activated catalytic subunit alpha 1) were among the list of bta-miR-486 target genes associated with this pathway (Fig. 1). Similarly, *FASN (*fatty acid synthase) and *G6PC2* (glucose-6-phosphatase catalytic subunit 2) were among the list of bta-miR-339a/b target genes (Supplementary Fig. S1). Also in this pathway, *G6PC*, *PYGM* (glycogen phosphorylase represented as *PYG*) and *PRKAR2B* (protein kinase cAMP-dependent type II regulatory subunit beta represented as *PKA*) were among the list of bta-miR-378 (Supplementary Fig. S2) target genes.

Based on the bta-miR-30b-5p target genes, the FoxO signaling pathway was associated with RFI (Supplementary Fig. S3). This pathway involves genes such as *G6PC3* (glucose-6-phosphatase catalytic subunit 3) and *SMAD3* (SMAD family member 3). Similarly, the bta-miR-339a/b target genes *G6PC2* and *TGFBR2* (transforming growth factor beta receptor 2) were also associated with this pathway (Supplementary Fig. S4). In addition, the Rap1 signaling pathway (Supplementary Fig. S5) was associated with RFI based on the bta-miR-423-5p target genes *FGFR1*, *FGFR2*, *FGFR17* (fibroblast growth factor receptor 1, 2 and 17, represented as *GF*), and *MAPKs* (mitogen-activated protein kinases).

### MiRNA and mRNA co-expression analysis

Weighted Gene Co-expression Network Analysis (WGCNA) approach was applied to investigate the role of regulatory networks related to RFI in skeletal muscle and liver of Nelore cattle. To this end, miRNA and mRNA expression data from genetically divergent RFI animals were used. To construct miRNA network, after quality control, 156 and 154 miRNAs expression data were used from skeletal muscle and liver, respectively. For mRNA network construction, after quality control, expression data (17,944 skeletal muscle and 16,076 liver mRNAs) from the same set of samples of this stutdy were used. Co-expression network analysis identified 13 feed efficient and five feed inefficient miRNA modules in skeletal muscle (Supplementary Fig. S6). The same approach applied to the skeletal muscle mRNA expression data, identified 34 feed efficient and 49 feed inefficient modules (Supplementary Fig. S7). From liver expression data, WGCNA identified 17 feed efficient and 22 feed inefficient miRNA modules (Supplementary Fig. S8), as well as 94 feed efficient and 118 feed inefficient mRNA modules were identified (Supplementary Fig. S9).

### Module-RFI relationship

The module-trait relationship analysis was carried out to study the correlation between RFI and the modules identified by WGCNA. To this end, Pearson’s correlations between miRNA module eigengenes (MEs) and RFI, and mRNA MEs and RFI were calculated.

In the skeletal muscle feed efficient group, four miRNA modules (Supplementary Fig. S10) and two mRNA modules (Supplementary Fig. S11) were significantly correlated with RFI (p-value < 0.10), while in the liver, four miRNA modules (Supplementary Fig. S13) and 12 mRNA modules (Supplementary Fig. S14) were found. In the feed inefficient group, no miRNA modules from the skeletal muscle (Supplementary Fig. S10) and eight mRNA modules (Supplementary Fig. S12) were significantly correlated with RFI, while in the liver, two miRNA modules (Supplementary Fig. S13) and 12 mRNA modules (Supplementary Fig. S15) were observed.

Table 4 shows the functional enrichment analysis (FDR < 0.10) obtained from WebGestalt software of MEs significantly correlated with RFI. Only those modules that had a significant functional enrichment of target genes (for miRNA modules) and genes (for mRNA modules) are presented in Table 4.

**Table 4:** Signaling pathways of miRNA and mRNA module eigengenes (MEs) that were significantly correlated with RFI for feed efficient and feed inefficient residual feed intake (RFI) groups.

RFI groups	MEs^a^	Corr^b^	p-value	S6ignaling Pathways	FDR^c^
**Skeletal muscle**					
**miRNA**					
Feed efficient	blue	−0.5	0.04	Insulin signaling pathway	1.44e-02
	purple	−0.7	0.008	FoxO signaling pathway	01.09e-03
	greenyellow	0.5	0.07	MAPK signaling pathway	7.39e-05
	yellow	−0.5	0.03	FoxO signaling pathway	9.89e-07
				Insulin signaling pathway	1.07e-05
**mRNA**					
Feed inefficient	saddlebrown	−0.5	0.09	Proteasome	8.62e-03
	pink	−0.5	0.06	Metabolic pathways	8.43e-02
	cyan	0.5	0.02	Inflammatory mediator regulation of TRP channels	7.33e-02
**Liver**					
**miRNA**					
Feed efficient	tan	−0.7	0.05	AMPK signaling pathway	1.11e-03
	pink	−0.8	0.01	Insulin signaling pathway	1.41e-04
	yellow	−0.8	0.006	AMPK signaling pathway	1.68e-04
	cyan	0.7	0.02	Insulin signaling pathway	6.8e-02
Feed inefficient	midnightblue	0.6	0.07	MAPK signaling pathway	7.69e-04
	darkgreen	0.6	0.03	Insulin signaling pathway	2.48e-06
**mRNA**					
Feed efficient	darkgrey	0.8	0.02	Oxidative phosphorylation	7.73e-03
	darkviolet	0.6	0.07	Complement and coagulation cascades	1.04e-02
	ivory	0.7	0.03	Primary immunodeficiency	9.81e-03
Feed inefficient	salmon	0.8	0.003	Protein processing	2.43e-02
	lightcyan	−0.6	0.07	Complement and coagulation cascades	3.86e-02
	saddlebrown	−0.7	0.02	Rap1 signaling pathway	5.82e-02

^a^miRNA and mRNA module eigengenes.

^b^Pearson’s correlations between module eigengenes and RFI.

^c^False discovery rate adjusted p-values by Benjamini-Hochberg methodology.

In the skeletal muscle feed efficient group, target genes of hub miRNAs from MEblue, MEpurple, MEgreenyellow, MEyellow, and liver hub miRNAs from MEtan, MEpink, MEyellow and MEcyan (liver tissue) were functionally enriched for insulin, FoxO, MAPK, and AMPK signaling pathways (Table 4). Three liver mRNA modules: MEdarkgrey, MEdarkviolet, and MEivory were functionally enriched for oxidative phosphorylation and immune system signaling pathways.

In the liver feed inefficient group, target genes of hub miRNAs into the MEmidnightblue, and MEdarkgreen were functionally enriched for MAPK and insulin signaling pathways. Three skeletal muscle mRNA modules: MEsaddlebrown, MEpink, and MEcyan, and three liver mRNA modules: MEsalmon, MElightcyan, and MEsaddlebrown, were functionally enriched for proteasome, metabolic pathways, inflammatory regulation, complement and coagulation cascades, and Rap1 signaling pathways.

### MiRNA: mRNA module interactions for RFI

Considering that miRNAs can negatively regulate mRNA abundance, miRNA MEs were correlated with mRNAs MEs from skeletal muscle in feed efficient (Supplementary Fig. S16) and feed inefficient (Supplementary Fig. S17) groups; and from liver in feed efficient (Supplementary Fig. S18) and feed inefficient (Supplementary Fig. S19) groups. Modules that were negatively correlated and with a p-value < 0.10 were selected for further investigation, however, due a large number of negatively correlated modules, Table 5 only shows negatively correlated modules that had the signaling pathways significantly enriched (FDR < 0.10).

**Table 5:** Signaling pathways of miRNAs module eigengenes (MEs) negatively correlated with mRNAs MEs for feed efficient and feed inefficient residual feed intake (RFI) groups.

RFI groups	miRNA MEs^a^	mRNA MEs^b^	Corr^c^	p-value	Signaling Pathways^d^	FDR^e^
**Skeletal muscle**						
Feed efficient	green	turquoise	−0.5	0.04	TGF-beta signaling pathway	2.33e-04
Feed inefficient	blue	tan	−0.7	0.002	Cytokine-cytokine receptor interaction -	5.01e-06
		salmon	−0.6	0.009	Inflammatory bowel disease	9.71e-02
	brown	tan	−0.6	0.03	Cytokine-cytokine receptor interaction	5.01e-06
	yellow	red	−0.5	0.05	Chemokine signaling pathway	1.61e-02
**Liver**						
Feed efficient	midnightblue	green	−0.8	0.02	Metabolism of xenobiotics by cytochrome P450	4.79e-03
		pink	−0.8	0.01	Metabolic pathways	9.15e-06
	turquoise	purple	−0.9	0.001	Rap1 signaling pathway	1.77e-04
		cyan	−0.8	0.02	VEGF signaling pathway	5.38e-03
Feed inefficient	pink	salmon	−0.7	0.03	Protein processing	2.43e-02
	magenta	salmon	−0.6	0.05	Protein processing	2.43e-02
	cyan	white	−0.8	0.003	Fatty acid metabolism	7.54e-02
	midnightblue	honeydew	−0.6	0.05	Steroid biosynthesis	8.43e-02
	yellow	green	−0.7	0.03	Proteasome	6.3e-06

^a,b^miRNA and mRNA module eigengenes.

^c^Pearson’s correlations between miRNA module eigengenes and mRNA module eigengenes.

^d^Signaling Pathways of miRNAs target genes present in mRNA MEs.

^e^False discovery rate adjusted p-values by Benjamini-Hochberg (1995) methodology.

In the skeletal muscle feed efficient group, one miRNA module (MEgreen) was negatively correlated to one mRNA module (MEturquoise), while in liver, two miRNA modules (ME midnightblue, and MEturquoise) were negatively correlated with four mRNA modules (MEgreen, MEpink, MEpurple, and MEcyan).

In the skeletal muscle feed inefficient group, three miRNA modules (MEblue, MEbrown, and MEyellow) were negatively correlated with three mRNA modules (MEtan, MEsalmon, and MEred). In the liver, five miRNA modules (MEpink, MEmagenta, MEcyan, MEmidnightblue, and MEyellow) were negatively correlated to four mRNA modules (MEsalmon, MEwhite, MEhoneydew, and MEgreen).

In order to make a more strong biological connection between miRNAs and mRNAs, we focused our functional enrichment analysis on the target genes from miRNA MEs present in mRNAs modules. In the skeletal muscle feed efficient group, target genes from mRNA modules were functionally enriched for signaling pathways related to TGF-beta. In liver, mRNA modules were functionally enriched for signaling pathways related to Rap1 and VEGF signaling pathways, metabolism of xenobiotics, and metabolic pathways (Supplementary Table S4).

In the skeletal muscle feed inefficient group, target genes from mRNA modules were functionally enriched for signaling pathways related to immune system. In the liver, mRNA modules were functionally enriched for signaling pathways related to protein and lipid metabolism (Supplementary Table S5).

**Figure 2:**
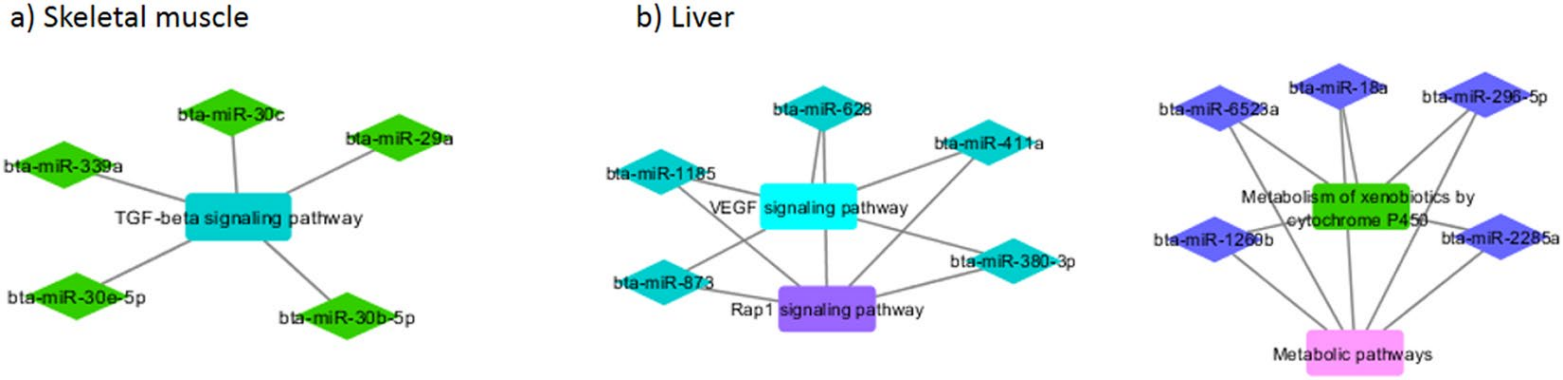
Co-expression networks of feed efficient group from skeletal muscle (**a**) and liver (**b**) in Nelore cattle. Colored diamonds represent the top 5 hub miRNAs within of each module, and colored round rectangles represents the signaling pathways associated (FDR ≤ 0.05) with the genes.

**Figure 3:**
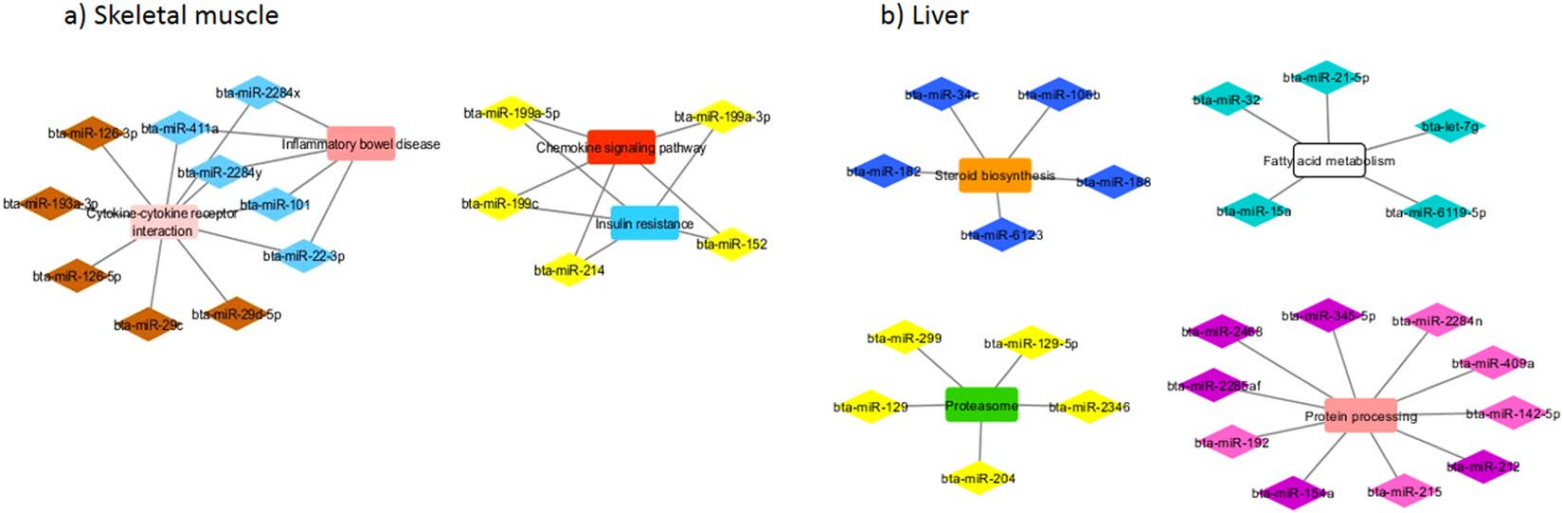
Co-expression networks of feed inefficient group from skeletal muscle (**a**) and liver (**b**) in Nelore cattle. Colored diamonds represent the top 5 hub miRNAs of each module, and coloured round rectangles represents the signaling pathways associated (FDR ≤ 0.05) with the genes.

In order to find key miRNAs involved in the co-expression networks and thereby, in the regulation of the phenotype, we selected the top 5 miRNAs based on greatest Module Membership (MM) values from miRNAs modules that were negatively correlated with mRNAs modules from skeletal muscle and liver feed efficient groups (Supplemental Table S6) and from skeletal muscle and liver feed inefficient groups (Supplemental Table S7). Figures 2 and 3 show co-expression networks among hub miRNAs and mRNA modules enriched for signaling pathways of feed efficient (Fig. 2) and feed inefficient (Fig. 3) groups from skeletal muscle (a) and liver (b) in Nelore cattle.

## Discussion

MiRNAs are important regulators of gene expression, presenting variation in their expression patterns in different cells and tissues. A single miRNA might regulate different targets, while a single transcript can be modulated by multiple miRNAs^18^, resulting in a complex regulatory network. To better understand the role of miRNAs in the regulation of RFI, differentially expressed (DE) miRNAs in the skeletal muscle and liver from feed efficient and feed inefficient Nelore cattle were identified. The DE bta-miR-486, found down-regulated in the skeletal muscle of feed efficient animals, has also been identified as down-regulated in feed efficient pigs and associated with skeletal muscle growth^2^. The DE bta-miR-339a/b and bta-miR-378 were also found as down-regulated in liver of feed efficient animals. The bta-miR-339a/b have been reported to be more expressed in bovine adipose tissue^19^; and miR-378a was found to affect lipid and xenobiotic metabolism, lipid storage and the glycolytic pathway. Moreover, miR-378a was reported to affect muscle development via regulation of myogenic repressor (MyoR) during myoblast differentiation^20^.

Among the target genes of bta-miR 486, we found some DE genes detected in previously skeletal muscle RNAseq study performed in this same population^10^: *ENAH*, *GAS7*, *ATF3*, *COL6A6*, and *C3*. The Activating Transcription Factor 3 (*ATF3*) is one of the transcription factors responsible for expression changes in this population. From bta-miR 378, the target genes *RGS2*, *ATP2A2*, *UCP2*, *COL1A1*, *UGGT1*, *FADS2*, *GLCE*, *RNASE6*, *SIX1* and *NPC2* were also DE in another previously liver RNAseq study performed in this population^11^. The genes Uncoupling protein 2 (*UCP2)* and Fatty acid desaturase 2 (*FADS2)* have a recognized role in carbohydrate and fatty acid metabolism and were found to be up-regulated in the feed efficient group. From bta-miR-30b-5p and bta-miR-339a/b target genes, are the DE genes *HSPB8*, *ATP2A2*, *SMAD1*, *NUFIP1*, *RGS2* and *G6PC3*, *SMAD3*, *TGFBR1*. From bta-miR 423-5p target genes, the genes *ATP2A2*, *IRF6*, *C1QC*, *SELL*, *COL1A1*, *FADS2*, *TGM2*, *FAM174B* and *GPX3* were also DE in the liver. The genes Regulator of G-protein signaling 2 (*RGS2)* and Collagen Type 1 (*COL1A1)* are related to body weight regulation, adiposity^21^ and skeletal system development^22^ and were found up-regulated in the feed efficient group.

Functional enrichment analysis of bta-miR-486, bta-miR-339a/b, and bta-miR-378 target genes indicated that the insulin signaling pathway was over-represented in both tissues. It is known that insulin and energy metabolism influences feed efficiency in cattle and pigs^23,24^, with higher levels of insulin and glucagon contributing to reduce feed intake^25^. Genes such as *PPARGC1A* and *AMPK*, present in this pathway, also have a recognized role in metabolic processes that influence feed efficiency in pigs^2^. PPARG coactivator 1 alpha is a key factor in mitochondrial biogenesis^26^ with a well known influence on lipid metabolism; while *AMPK* is a basic regulator of cellular and body energy metabolism and may enhance activity of mitochondrial proteins of oxidative metabolism^27^.

In a recent integrative analysis performed with samples of this Nelore population, Oliveira *et al*.^28^ identified the *PPARGC1A* as a target gene for a downregulated miRNA in animals with low intramuscular fat deposition. It has been shown by some studies that feed efficient animals tends to be leaner, with lower intramuscular fat deposition^29,30^. These results reinforce the role of *PPARGC1A* on fat metabolism and suggest a possible role of *PPARGC1A* on feed efficiency metabolism of Nelore cattle. By assuming that *PPARGC1A* and *AMPK* protein levels are up-regulated, as bta-miR-486, bta-miR-339a/b, and bta-miR-378 are down-regulated, we can postulate that these genes may contribute to higher feed efficiency. A word of caution is necessary in this conclusion since, although the canonical effect of miRNAs on gene expression is mRNA downregulation^31^, some studies have reported that positive miRNA regulation is also common; which we also observed in our study from weighted gene co-expression analysis. Positive correlations may reflect secondary miRNA targets^13^ or an adaptive target miRNA response^14^. Here, we considered the miRNA-mRNA down-regulation as the primary effect, however, it is important to note that our study is based on *in silico* analysis.

We also applied the WGCNA of miRNA and mRNA expression data to investigate miRNA interactions with expressed genes previously identified in this same population of animals^10,11^. WGCNA relies on the assumption that strongly correlated expression levels of genes indicate that those genes work cooperatively and thereby contribute to the phenotype^23^. By relating modules to external trait, we were willing to identify modules that are significantly correlated with RFI. Our results indicate that four miRNA modules significantly correlated with RFI (MEblue, MEyellow, MEpink, and MEcyan, from both tissues), were enriched for the insulin signaling pathway in feed efficient animals. Interestingly, the bta-miR-486 is one of the top five hub miRNAs in MEblue; from which we can infer a potential role of bta-miR-486 on genes acting on the insulin signaling pathway. Taken together, these results indicate that regulation of insulin metabolism by the DE miRNAs described here might play a role in feed efficiency of Nelore cattle.

The DE miRNAs bta-miR-30b-5p and bta-miR-339a/b were also down-regulated in feed efficient animals. The bta-miR-30-5p has been reported to inhibit bovine muscle cell differentiation^32^. Thus, if one considers downregulation of bta-miR-30-5p as predictive of up-regulated genes for muscle development, it would be a putative mechanism for the association of bta-miR-30b-5p with feed efficiency. Skeletal muscle growth has been suggested as a potential strategy for the improvement of feed efficiency in pigs^2^ and bovines; as in general; feed efficient animals presents greater *Longissimus* muscle area, suggesting greater muscle deposition^30^. Although no difference was observed between divergent RFI groups in our study, increased REA was observed for feed efficient animals when analyzing a larger sample of this Nelore population (N = 575)^33^, supporting our hypothesis.

Enrichment analysis of bta-miR-30b-5p and bta-miR-339a/b target genes revealed that the FoxO signaling pathway was over-represented spotlighting its possible role in Nelore cattle feed efficiency. FoxO transcription factors regulate gene expression of relevant physiological events including glucose metabolism and resistance to oxidative stress^34^. Pathways related to response to oxidative stress were previously associated to feed efficient animals of this population. These authors found that feed efficient animals presented the NRF2-regulated signaling pathway upregulated, which protects mitochondria from oxidative stress during fasting^11^. Therefore, the down-regulated bta-miR-30b-5p and bta-miR-339a/b suggest that the FoxO signaling pathway genes are activated, which may contribute to a higher feed efficiency in Nelore cattle. In addition, from WGCNA results, two miRNA modules (MEpurple and MEyellow) significantly correlated to RFI, were also enriched for FoxO signaling pathway in feed efficient animals.

The Rap1 signaling pathway contains bta-miR-423-5p target genes such as *FGFR1* and *MAPK12*. Based on WGCNA results, the miRNA MEgreenyellow was enriched for MAPK Signaling pathway, and mRNA MEpurple was enriched for Rap1 signaling pathway in feed efficient animals. Rap1 is a protein that controls MAP kinase activity^35^ and acts as a regulator of the storage of nutrients in the white adipose tissue and skeletal muscle^24^. These enzymes stimulate the secretion of leptin, an adipokine that can suppress appetite in rodents^36^ and has been shown to play major roles in the regulation of body weight and feed intake in bovines^37^, which could explain the Rap1 signaling pathway association with higher feed efficiency.

Regarding the miRNA:mRNA module interactions, other important biological functions related to feed efficiency were highlighted. Co-expression analyses of miRNA and mRNA muscular expression data in feed inefficient animals revealed gene sets that shared functional enrichment for immune system modules (MEtan, MEsalmon, and MEred; Fig. 2a). Cytokines are crucial regulators involved in adaptive inflammatory host defenses^38^. Inflammation and immune response biological processes have already been associated with lower feed efficiency in cattle, with feed inefficient animals presenting increased liver lesions, possibly due to altered lipid metabolism or bacterial infection resulting from a higher feed intake^29^. Among hub miRNAs from MEbrown and MEyellow, predicted to target these immune system modules, the bta-miR 29 and bta-miR-199a-3p target genes functions were related to hepatic system disease^3^, oxidative stress and immune system in bovines^39^. Tizioto *et al*.^10^ found genes involved in oxidative stress and in antioxidant mechanisms down-regulated in feed inefficient animals of this population, supporting our findings regarding the immune system-related modules in this group. In addition, hub miRNAs, such as bta-miR 29b, bta-miR 101, bta-miR-193a-3p, and bta-miR-126-5p were already reported to be up-regulated in feed inefficient cattle^3^. Moreover, two mRNA modules (MEcyan and MElightcyan) significantly correlated with RFI were enriched for innate immunity and thermo inflammatory regulation processes. Thermoregulation is another physiological process likely to contribute to variation in RFI in cattle^40^.

From liver of feed inefficient animals, hub miRNAs from two modules (MEcyan and MEmidnightblue, Fig. 2b) target mRNA modules enriched for fatty acid metabolism (MEwhite) and steroid biosynthesis (MEhoneydew). In bovine, many miRNAs, such as hub miRNAs from MEcyan (bta-miR-7, bta-miR15a, bta-miR-21-5p) and MEmidnightblue (bta-miR-106b) have been previously associated to adipose tissue^19^ and feed efficiency^41^. Oliveira *et al*.^28^ identified the bta-let-7f and bta-let-7a-5p as down-regulated in animals with different amount of intramuscular fat deposition; which indicates that these miRNAs may be involved in adipose tissue development^6^. In addition, Tizioto *et al*.^11^ found genes responsible for lipid synthesis up-regulated in feed inefficient animals and concluded that gene expression between feed efficient and feed inefficient cattle are related to lipid catabolism, which support our findings of lipid metabolism modules in this group.

Additionally, in feed inefficient animals, hub miRNAs from MEpink and MEmagenta targeted two mRNA modules (MEsalmon, MEgreen) that were enriched for protein processing and proteasome pathways. The proteasome degradation pathway is essential for many cellular processes, including immune system and responses to oxidative stress^42^. The implications of oxidative stress in major processes underlying variation in feed efficiency in this population has been recently addressed^10^. Between hub miRNAs from MEyellow, the bta-miR-299 and bta-miR-204 target genes function was related to apoptosis^32^ and heat stress in bovine^39^, respectively.

In feed efficient animals, skeletal muscle hub miRNAs from MEgreen were correlated with mRNA MEturquoise, which was enriched for TGF-beta signaling pathway (Fig. 3b). The transforming growth factor-beta (TGF-β) signaling pathway is a potent negative regulator of skeletal muscle growth and development^43^. Jing *et al*.^2^ found the TGF-β signaling pathway down-regulated in feed efficient pigs and reported that miR-29 and miR-30b, hub miRNAs in the MEgreen module, are both inhibitors of TGF-beta and are up-regulated in more feed efficient pigs. As discussed above and supporting our findings, the skeletal muscle growth biological process is associated with a higher feed efficiency. Taken together, these results further suggest the role of these miRNAs in the regulation of muscle development and feed efficiency in Nelore cattle.

From liver of feed efficient animals (Fig. 3a), hub miRNAs clustered to the MEmidnightblue had targets on mRNA MEpink; enriched for metabolic pathways; and on mRNA MEgreen; enriched for metabolism of xenobiotics by cytochrome P450. Consistent with the biology of feed efficiency, a wide range of biological processes are related to metabolic pathways such as energy, protein, carbohydrate, lipid and the xenobiotics metabolism; which were previously reported as up-regulated in feed efficient animals of this population^10^. Among hub miRNAs from MEmidnightblue, the bta-miR-2285 has been shown to be expressed in bovine immune tissue^7^ and bta-miR 18a and bta-miR 296 have been reported with a role in lactose metabolism^28^ and angiogenesis^44^. Additionally, hub miRNAs (bta-miR 628, bta-miR 1185, bta-miR 873, bta-miR 411a and bta-miR 380-3p) from another module (MEturquoise) were correlated with mRNA MEcyan, which was enriched for VEGF signaling pathway. The Vascular Endothelial Growth Factor (VEGF) is a key regulator of angiogenesis and vascularization^44^ and alterations in angiogenesis may have implications on feed efficiency, as vascularity of the intestine is crucial for nutrient transport and absorption^45^. Alexandre *et al*.^29^ reported differentially co-expressed genes related to angiogenesis between divergent feed efficiency groups in cattle.

Muscle and liver tissues have potential roles in the control of variation in feed efficiency. Muscle is a major user of energy within the body. Furthermore, liver is a central controller of metabolism and animal oxygen consumption^3^. Several studies have investigated the role of these tissues in feed efficiency, however, as far as we know there are no reports of miRNAs-mRNA feed efficiency co-expression networks. The functional analysis of DE miRNA’s target genes and miRNA- mRNA correlated modules in skeletal muscle and liver has revealed that primarily insulin, muscle development, lipid, and immune system signaling pathways seems to influence RFI in this population of Nelore cattle. Some of the signaling pathways found in this study have already been related to feed efficiency, however, the miRNA-mRNA interaction is new and may help to elucidate important unknown regulatory mechanisms of feed efficiency in Nelore cattle.

The differential expression analysis showed that the DE bta-miR-486 and the insulin signaling pathway might have a potential role in the variation of RFI in Nelore cattle. The bta-miR-30b-5p, bta-miR-339a/b, bta-miR-378 and bta-miR-423-5p were DE and may also have potential roles in pathways related to feed efficiency. Also, some important feed efficiency DE genes were found as target genes of these DE miRNAs. Our integrative network analysis shows that hub miRNAs, such as bta-miR-7g, bta-miR15a, bta-miR-21, bta-miR 29, bta-miR-30b, bta-miR-106b, bta-miR-199a-3p, bta-miR-204, and bta-miR 296 can potentially interact with several genes related to oxidative stress, metabolism of xenobiotics, fatty acid metabolism and muscle development, for which functions have already been discussed. Hub miRNAs are the highly interconnected nodes in a network, and may act as potential regulators in co-expressed networks^13^. However, while some hub miRNAs have biological functions described in the literature, some miRNAs as bta-miR 1185, have no documented functions. It is possible that those unknown miRNAs may be involved in the same pathways as the known miRNAs and thereby, in the regulation of specific functions or pathways related to feed efficiency in Nelore cattle.

Overall, the miRNA-mRNA co-expression networks associated with feed inefficient animals were related to immune system, oxidative stress and lipid metabolism, while for feed efficient animals besides being related to skeletal muscle growth and development, they were also related to metabolism of xenobiotics and fatty acid metabolism. Biological processes of fatty acid biosynthesis are down-regulated in feed inefficient animals, while pathways related to muscle growth, response to oxidative stress and xenobiotics metabolism seems to be up-regulated in feed efficient animals of this population^10,11^. This study reinforces the role of these pathways in feed efficiency biology and indicates a possible new role of miRNAs in the regulation of this phenotype, as revealed by differential expression and WGCN analysis.

Since feed efficiency is a relevant economic trait and a complex variable with many physiological components, it is substantial to investigate the miRNA-mRNA regulation, and thereby identify the possible key drivers of this phenotype. Although no functional experiment was done to support the miRNAs expression levels and the implication of these miRNAs on the phenotypes, these are the primary regulators based on the methodology; bioinformatics analysis and its combination with obtained datasets; to indicate mechanisms of regulation and signaling pathways involved with feed efficiency of Nelore cattle.

In the present work, miRNA-mRNA regulatory networks and hub miRNAs related to RFI were identified by miRNA differential expression and weighted gene co-expression network analysis. Important signaling pathways, such as insulin and muscle development were highlighted, as well the role of some key miRNAs, as bta-miR 485, bta-miR-7, bta-miR15a, bta-miR-21, bta-miR 29, bta-miR-30b, bta-miR-106b, bta-miR-199a-3p, bta-miR-204, and bta-miR 296, that may regulate biological pathways related to feed efficiency. This study provides new evidences for miRNA regulation in feed efficiency biology of Nelore cattle and thereby presents potential targets for improving the efficiency of beef production.

## Material and Methods

### Ethics statement

Experimental procedures were carried out in accordance with the relevant guidelines provided by the Institutional Animal Care and Use Committee Guidelines of the Embrapa Pecuária Sudeste – Protocol CEUA 01/2013. The Ethical Committee of the Embrapa Pecuária Sudeste (São Carlos, São Paulo, Brazil) approved all experimental protocols (approval code CEUA 01/2013).

### Phenotypic and expression data

Description of phenotypic data and genomic heritability for RFI (kg/day), intramuscular fat (IMF; %) and ribeye muscle area (REA; cm^2^) from Nelore steers were previously reported; with mean values of 0.001 ± 0.62 and 0.33 for RFI^9^, 2.77 ± 0.06 and 0.29 for IMF^46^ and 59.98 ± 7.55 and 0.27 for REA^47^. The genomic heritability is the estimated proportion of phenotypic variance explained by genome markers, as described in De Oliveira *et al*.^9^.

BLUP estimates of genetic merit for RFI were generated for 585 Nelore steers. However, liver Nelore samples were available for only 83 of animals, which were ranked according to their aditive genetic merit for RFI to select 20 animals genetically divergent. Where possible animals that had common sires were sampled only when they belonged to different tails of the BLUP distribution^11^. Residual feed intake phenotypes from this population were previously used to perform a RNA-seq study of skeletal muscle^10^ and liver^11^ in which steers were sampled to obtain low RFI or feed efficient animals (N = 10) and high RFI or feed inefficient animals (N = 10), with RFI mean values of −0.6832 (Kg/day) and 0.5296, respectively (Table 1). In total, 18,332 genes were expressed in skeletal muscle, and 16,962 genes were expressed in liver. These genes were used for gene co-expression analysis.

### MiRNA expression data

The skeletal muscle and liver cDNA libraries preparation were conducted in the Laboratory Multiuser Esalq in Piracicaba/SP/Brazil, according to the protocol described by Illumina, available in http://genome.med.harvard.edu/documents/illumina/TruSeq-SmallRNA-SamplePrep-Guide-15004197-A.pdf. The sequencing of cDNA libraries was conducted on a MiSeq (Illumina, San Diego, CA) with Miseq Reagent Kit V3 150 cycles. All of the procedures and standards were performed according to the manual of Miseq Reagent Kit.

The FastQC tools (http://www.bioinformatics.babraham.ac.uk/projects/fastqc) and FASTX (http://hannonlab.cshl.edu/fastx-toolkit) were used to check the quality of reads according to the following the parameters: [-q 28] = Minimum quality score to keep; [-p 70] = Minimum percent of bases that must have [-q] quality. Reads with non-canonical letters or with low quality were removed, the 3′ adapters were trimmed and sequences shorter than 18 nt were discarded. After quality control, reads were subjected to alignment to bovine genome reference UMD version 3.1 (Ensembl 84: Mar 2016) through the software miRDeep2 version 2.0.0.7^48^. The user input parameters used to run miRDeep2 were: length of miRNAs is set at 18 nucleotides; low-quality reads are filtered out at the alignment stage; read with less than 20 phred score was filtered out; multi-mapping reads with alignments to more than 100 genomic loci were filtered out; reads mapping to tRNA, sno-RNA and piRNA were also excluded and the score value for the potential miRNA precursor that was previously described was -10 (default). The reads were then mapped to regions of the genome using the Bowtie tool^49^, built in miRDeep2 software. The mature.bta.fa, hairpin.bta.fa and mature.hsa.fa files were extracted from miRBase (http://www.mirbase.org/ftp.shtml). The mature.bta.fa and hairpin.bta.fa are fasta format sequences of all mature miRNA sequences and all miRNA hairpins of *Bos taurus* species, respectively. The mature.hsa.fa are fasta format sequences of all mature miRNA sequences of *Homo sapiens* species.

### Differentially expressed miRNAs

For the differential expression analysis, in order to generate differentiated groups, we reduced the sample size to 8 libraries per tissue. So, differentially expressed (DE) miRNAs were identified from eight small RNA libraries derived from skeletal muscle (N = 4 [low RFI or feed efficient]; N = 4 [high RFI or feed inefficient]) and eight small RNA libraries derived from liver (N = 4 [low RFI or feed efficient]; N = 4 [high RFI or feed inefficient]) separately. The read count data was filtered as follows: i) miRNAs with zero counts were removed; ii) miRNAs for which less than 1/5 of samples have 0 counts were removed. After filtering, expressed miRNAs from skeletal muscle and liver tissue were analyzed for differential expression using the “nbinomTest” function of DESeq. 2^50^. The Benjamini-Hochberg method^51^ to control for the rate of false positive (FDR) of 10% was used for the determination of miRNAs differentially expressed between feed efficient and feed inefficient groups.

The FDR threshold was defined *a priori* based on the experimental design. Also, due to the number of genes and miRNAs tested, we set an FDR threshold of 0.1 (*i*.*e*., 10% of false positives are expected) to correct for false positive albeit avoiding losing too much information, as these are exploratory analyses that should indicate biological responses to be further verified.

### MiRNA target predictions and functional enrichment analysis

The target genes of DE miRNAs from skeletal muscle and liver were predicted with TargetScan^52^ and miRanda^53^ software. The TargetScan (Release 7.2) predicts biological targets of miRNAs by searching for the presence of conserved 8mer, 7mer, and 6mer sites^52^. The conserved miRNAs family threshold was used, and the search was performed for mammals and customised by species (cow/Bostaurus). miRanda is a software for target site identification from sequence information^53^. It compares the miRNAs complementarity to 3′ UTR regions of the genome. Using a perl script we generated a fasta file with all 3′ UTR regions of Ensembl 84: Mar 2016 bovine genome (UMD 3.1) to use as input in miRanda. After this first approach and in order to predict the potential regulatory target transcripts, the target genes were filtered by skeletal muscle^10^ and liver^11^ mRNA expression data previously analysed on the same set of samples. Functional enrichment analysis of target genes was performed by WebGestalt^17^ using *B*. *taurus* as the organism of interest, and the Overrepresentation Enrichment Analysis (ORA) as the method of interest.

### mRNA expression data

The processing and analysis of mRNA expression data from skeletal muscle and liver from the same population of animals (N = 20) used in this study were described in Tizioto *et al*.^10^ and Tizioto *et al*.^11^, respectively. The sample accession of mRNA expression data is PRJEB15314, as described in Tizioto *et al*.^10^.

### Co-expression analysis of miRNA and mRNA expression data

Co-expression networks were constructed by WGCNA^54^ v1.36 package in RStudio environment using miRNA and mRNA expression data from 20 libraries per tissue: miRNA (N = 10 low RFI or feed efficient; N = 10 high RFI or feed inefficient) and mRNA (N = 10 low RFI or feed efficient; N = 10 high RFI or feed inefficient) skeletal muscle and liver expression data.

MiRNA and mRNA network construction were done separately for feed efficient and feed inefficient groups. MiRNA network construction and module detection used the step-by-step network construction with a soft threshold of β = 6 (R^2^ > 0.90) and a minimum module size of 5. mRNA network construction used the step-by-step network construction with a soft threshold of β = 6 (R^2^ > 0.91) and a minimum module size of 30.

Five was chosen as the minimum module size for the miRNAs due to the smaller size of the miRNA transcriptome relative to the mRNA transcriptome^53,54^.The topological overlap distance calculated from the adjacency matrix is then clustered with the average linkage hierarchical clustering. The default minimum cluster merge height of 0.25 was retained.

### Relating modules to external trait (RFI)

The Module-Trait relationships were estimated by calculating the Pearson’s correlations between the module eigengenes and the animals’ phenotypic information to select potential biologically interesting modules that could explain the phenotypic differences between groups. WGCNA modules of miRNA and mRNA were considered significantly correlated with RFI when correlation p-values between MEs and RFI were <0.10. miRNA modules significantly correlated with RFI were then explored to identify the hub miRNAs, which are the miRNAs that have higher connectivity inside the module and are probably more informative^12^. Hub miRNAs were selected based on the top 5 greatest Module Membership (MM) values.

### MiRNA: mRNA module interactions

An integrative analysis was performed correlating miRNAs MEs with mRNAs MEs from skeletal muscle and liver for feed efficient and feed inefficient groups separately. Modules with negative correlation and a p-value < 0.10 were selected for functional enrichment analysis.

### Functional enrichment analysis of correlated modules

To better understand the biological significance of the modules identified, the functional enrichment analysis of target genes and genes was performed by WebGestalt^17^ web tool. The functional enrichment analysis used the list of target genes of hub miRNAs from correlated miRNA modules overlapped with genes from correlated mRNAs modules. Co-expression networks among hub miRNAs and the GO terms of the target genes inside mRNAs correlated modules were constructed in Cytoscape v.3.3.0 0^55^.

## Electronic supplementary material

Supplementary Table S1

Supplementary Table S2

Supplementary Table S3

Supplementary Table S4

Supplementary Table S5

Supplementary Table S6

Supplementary Table S7

SupplementaryInformation

## Data Availability

The sample accession of miRNA expression data from skeletal muscle and liver in ENA repository (EMBL-EBI) is PRJEB23965.
