# Supplementary material for: An integrative transcriptome analysis indicates regulatory mRNA-miRNA networks for residual feed intake in Nelore cattle
Source: Sci Rep. 2018 Nov 20;8:17072. doi: 10.1038/s41598-018-35315-5 (PMC6244318; doi:10.1038/s41598-018-35315-5)
Supplement: Supplementary file 8 — SupplementaryInformation [file 41598_2018_35315_MOESM8_ESM.docx]

**An integrative transcriptome analysis indicates regulatory mRNA-miRNA networks for residual feed intake in Nelore cattle**

Priscila S.N. de Oliveira^1^, Luiz L. Coutinho^2^, Polyana C. Tizioto^3^, Aline S.M. Cesar^2^; Gabriella B. de Oliveira^2^, Wellison J. da S. Diniz^4^, Andressa O. de Lima^4^; James M. Reecy^5^, Gerson B. Mourão^2^, Adhemar Zerlotini^6^ and Luciana C.A. Regitano^1*^

^1^Embrapa Pecuária Sudeste, São Carlos, SP, 13560-970, Brazil

^2^Department of Animal Science, University of São Paulo, Piracicaba, SP, 13418-900,

Brazil.

^3^NGS Genomic Solutions, Piracicaba, SP, 13418-900, Brazil.

^4^Department of Genetics and Evolution, Federal University of São Carlos, São Carlos, SP, 13565-905, Brazil.

^5^Department of Animal Science, Iowa State University, Ames, IA, 50011, USA.

^6^ Embrapa Informática Agropecuária, Campinas, SP, 770901, Brazil.

**Supplementary information**

**Supplementary Figures:**


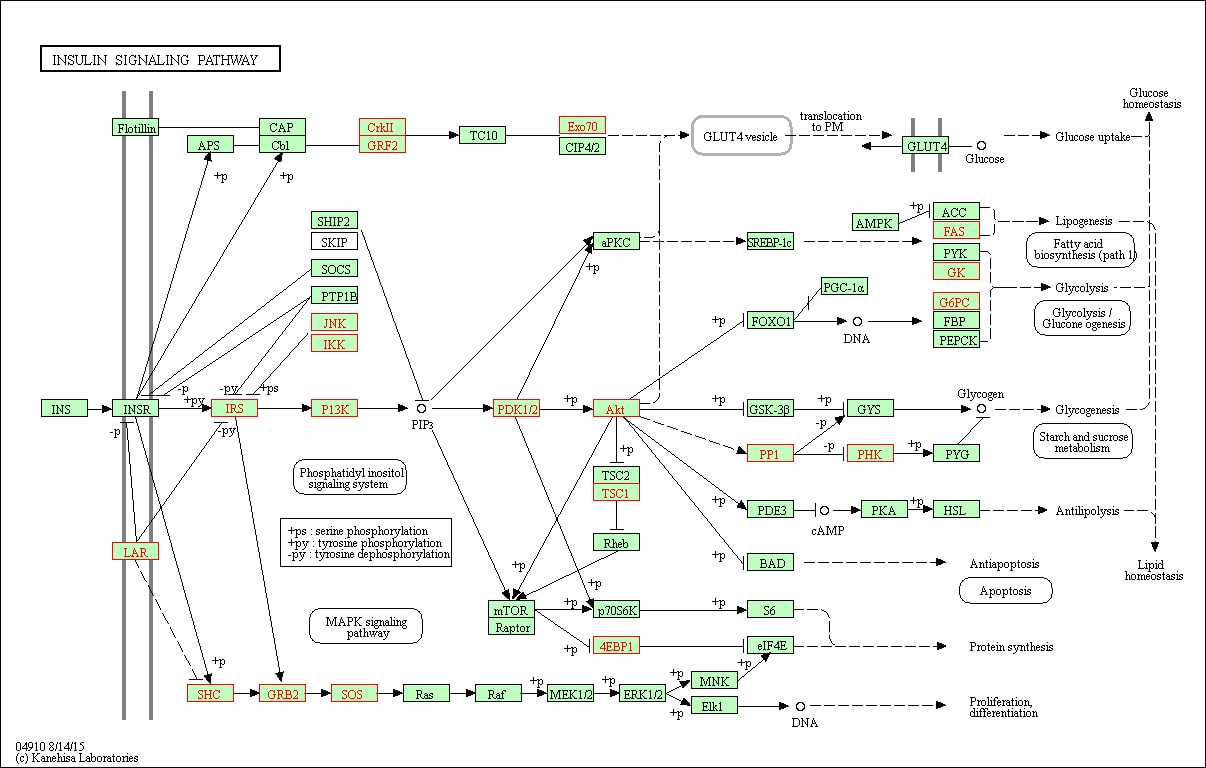


**Supplementary Figure S1**. KEGG^18^ Insulin Signalling pathway identified by WebGestalt from the bta-miR-339a/b target gene list. Boxes with red labeling indicates target genes for the DE miRNAs, while boxes with black labeling are not targeted genes. Solid lines mean direct interaction and dashed lines an indirect interaction between genes.


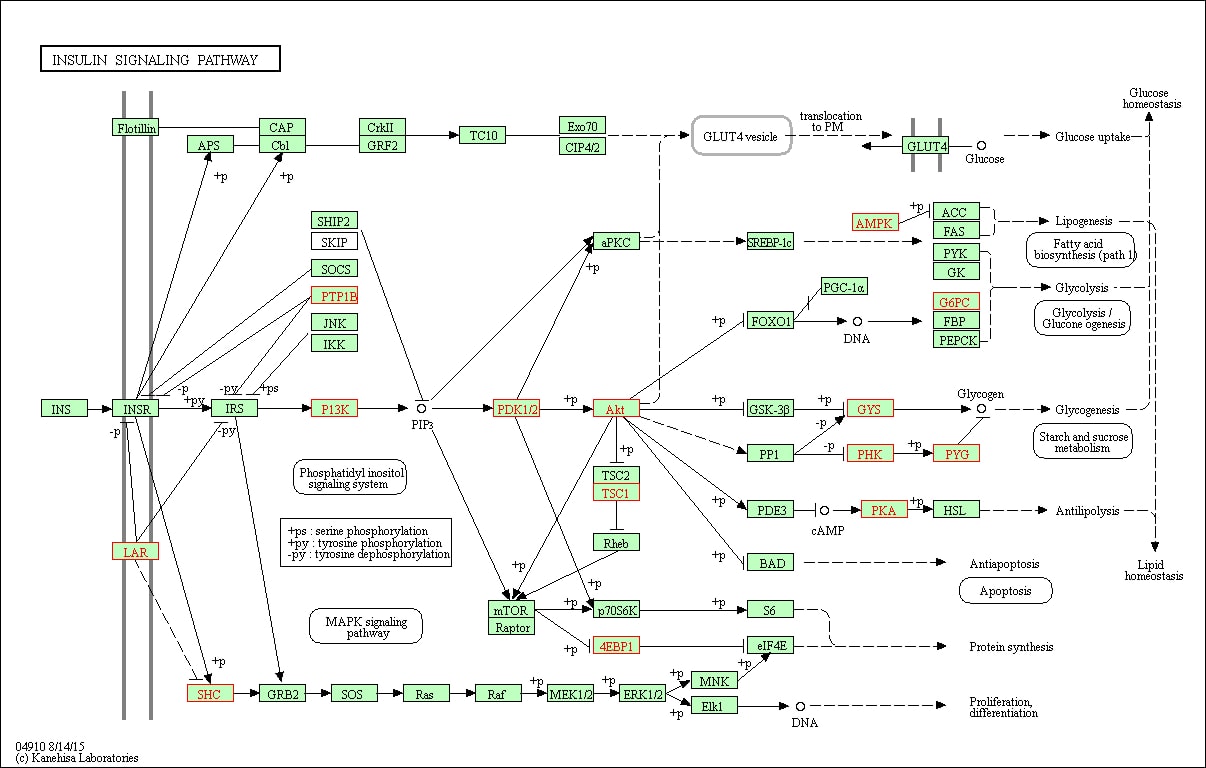


**Supplementary Figure S2**. KEGG^18^ Insulin Signalling pathway identified by WebGestalt from the bta-miR-378 target gene list. Boxes with red labeling indicates target genes for the DE miRNAs, while boxes with black labeling are not targeted genes. Solid lines mean direct interaction and dashed lines an indirect interaction between genes.


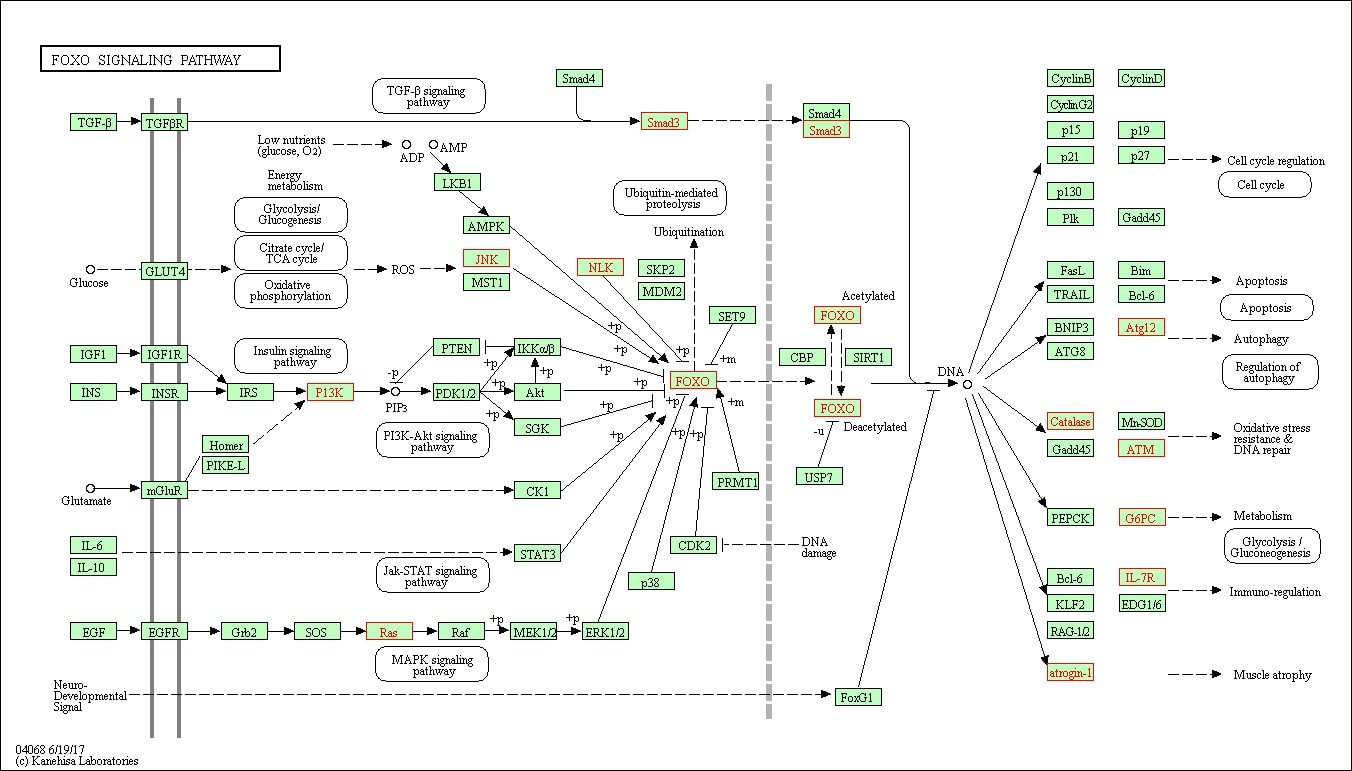


**Supplementary Figure S3**. KEGG^18^ FoxO Signalling pathway identified by WebGestalt from the bta-miR-30b-5p target gene list. Boxes with red labeling indicates target genes for the DE miRNAs, while boxes with black labeling are not targeted genes. Solid lines mean direct interaction and dashed lines an indirect interaction between genes.


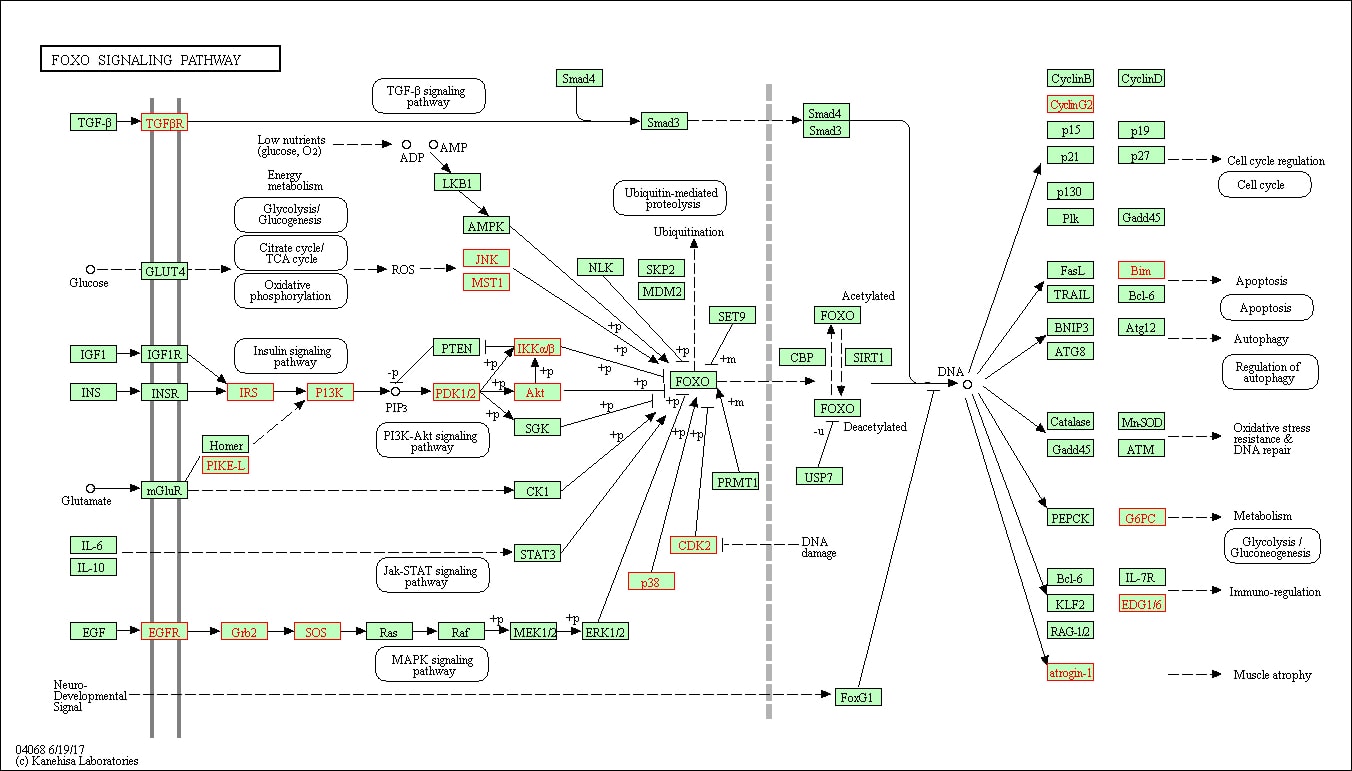


**Supplementary Figure S4**. KEGG^18^ FoxO Signalling pathway identified by WebGestalt from the bta-miR-339a/b target gene list. Boxes with red labeling indicates target genes for the DE miRNAs, while boxes with black labeling are not targeted genes. Solid lines mean direct interaction and dashed lines an indirect interaction between genes.


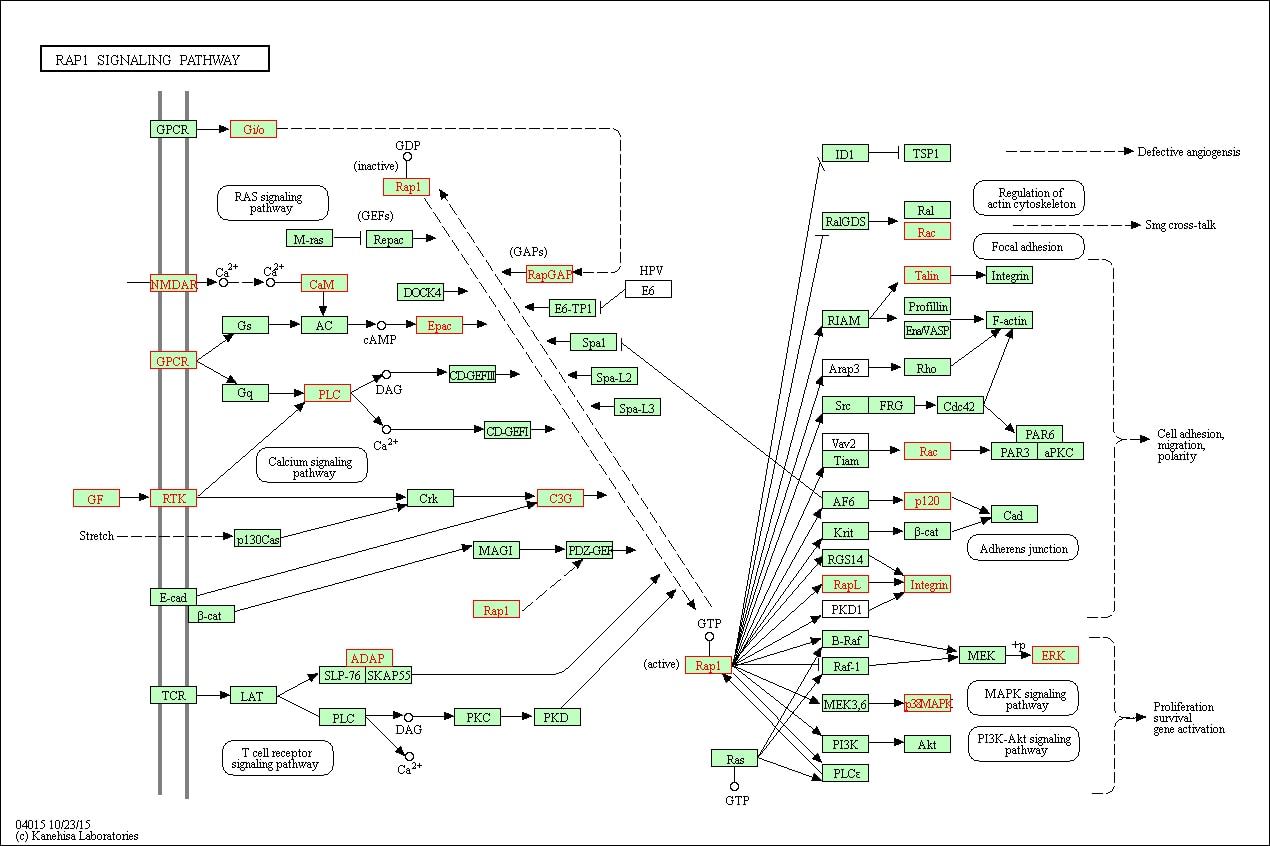


**Supplementary Figure S5**. KEGG^18^ Rap1 Signalling pathway identified by WebGestalt from the bta-miR-423-5p target gene list. Boxes with red labeling indicates target genes for the DE miRNAs, while boxes with black labeling are not targeted genes. Solid lines mean direct interaction and dashed lines an indirect interaction between genes.


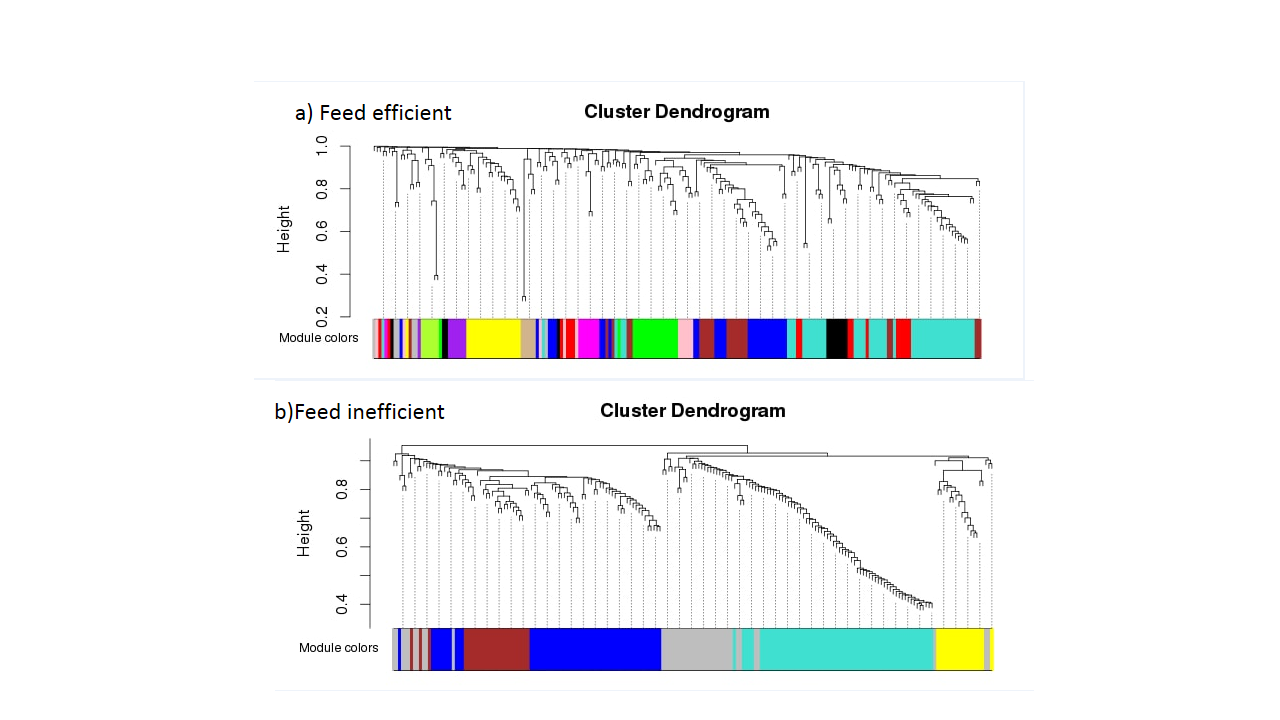


**Supplementary Figure S6.** Cluster dendogram of co-expression network analysis from a) Feed efficient and b) Feed inefficient miRNA modules in skeletal muscle.


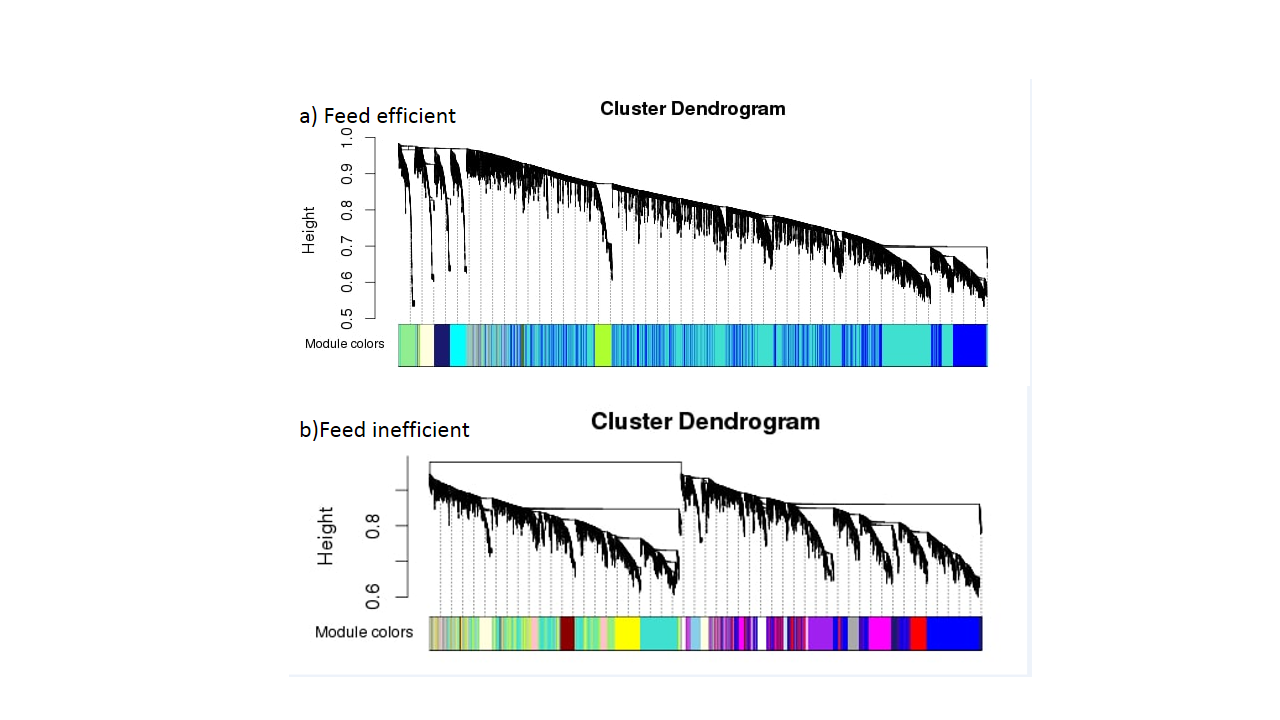


**Supplementary Figure S7.** Cluster dendogram of co-expression network analysis from a) Feed efficient and b) Feed inefficient mRNA modules in skeletal muscle.


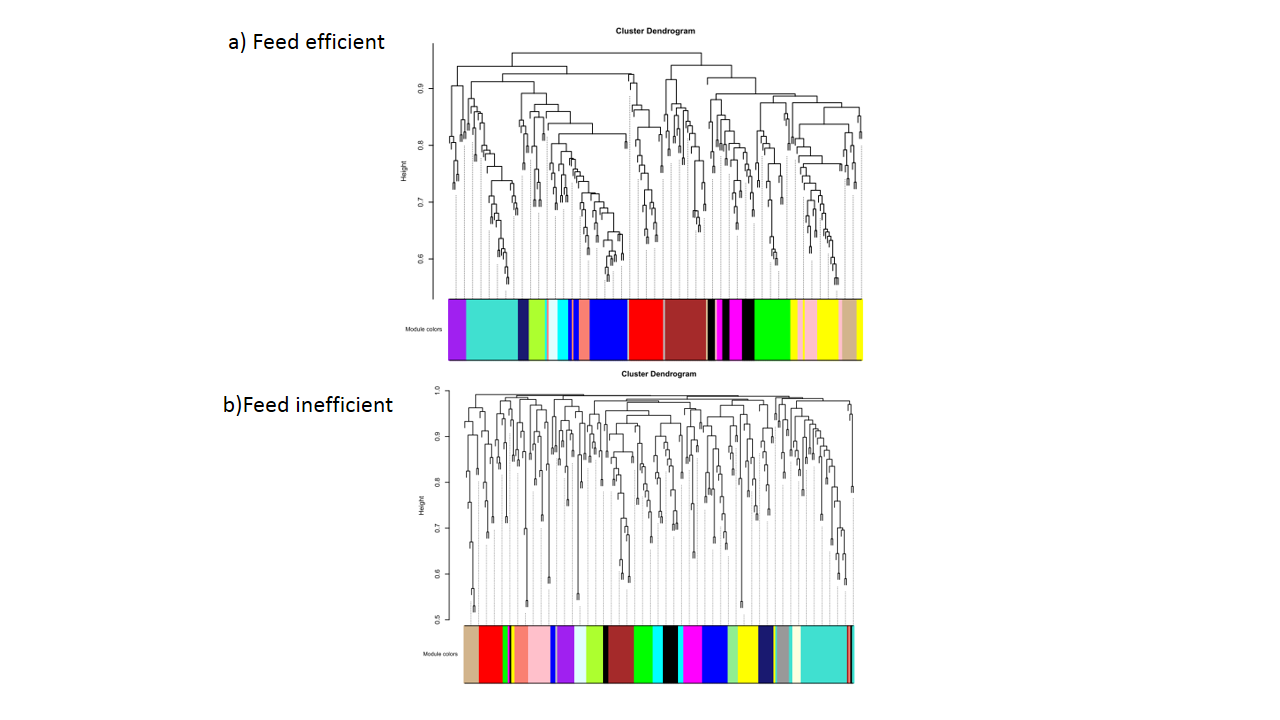


**Supplementary Figure S8.** Cluster dendogram of co-expression network analysis from a) Feed efficient and b) Feed inefficient miRNA modules in liver.


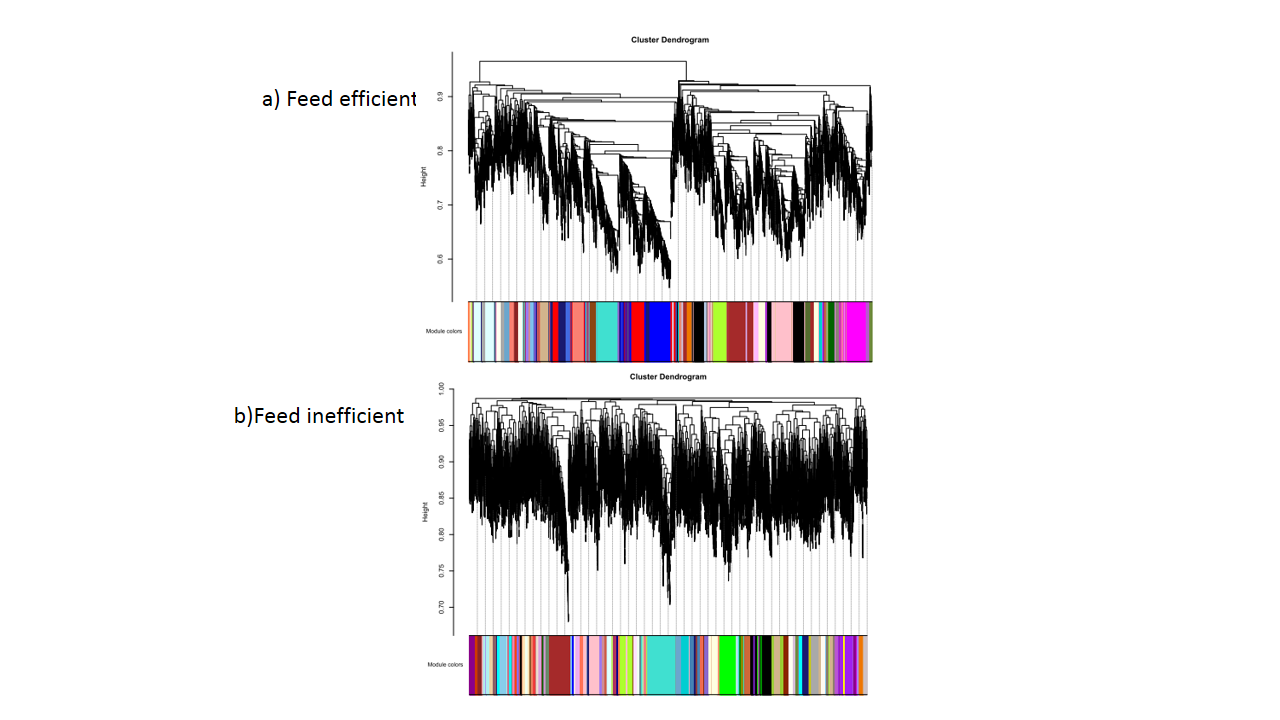


**Supplementary Figure S9.** Cluster dendogram of co-expression network analysis from a) Feed efficient and b) Feed inefficient mRNA modules in liver.


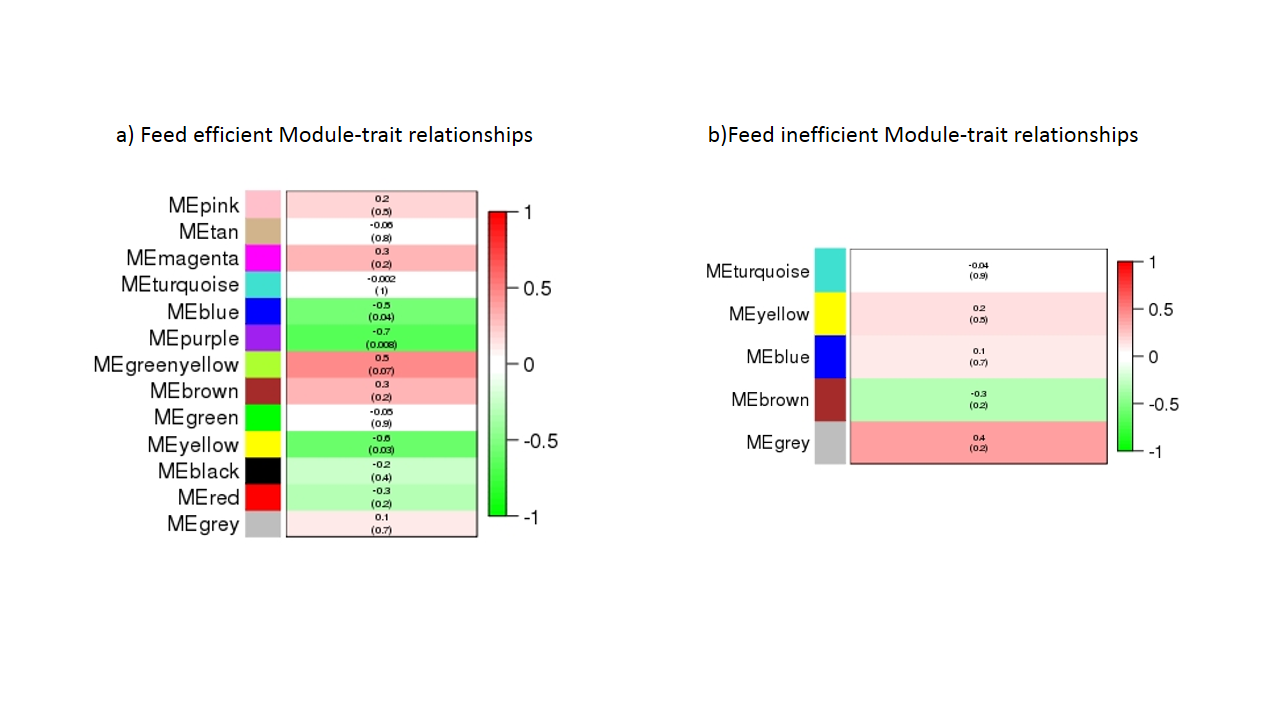
**Supplementary Figure S10.** Module-trait relationship from a) Feed efficient and b) Feed inefficient miRNA modules in skeletal muscle.


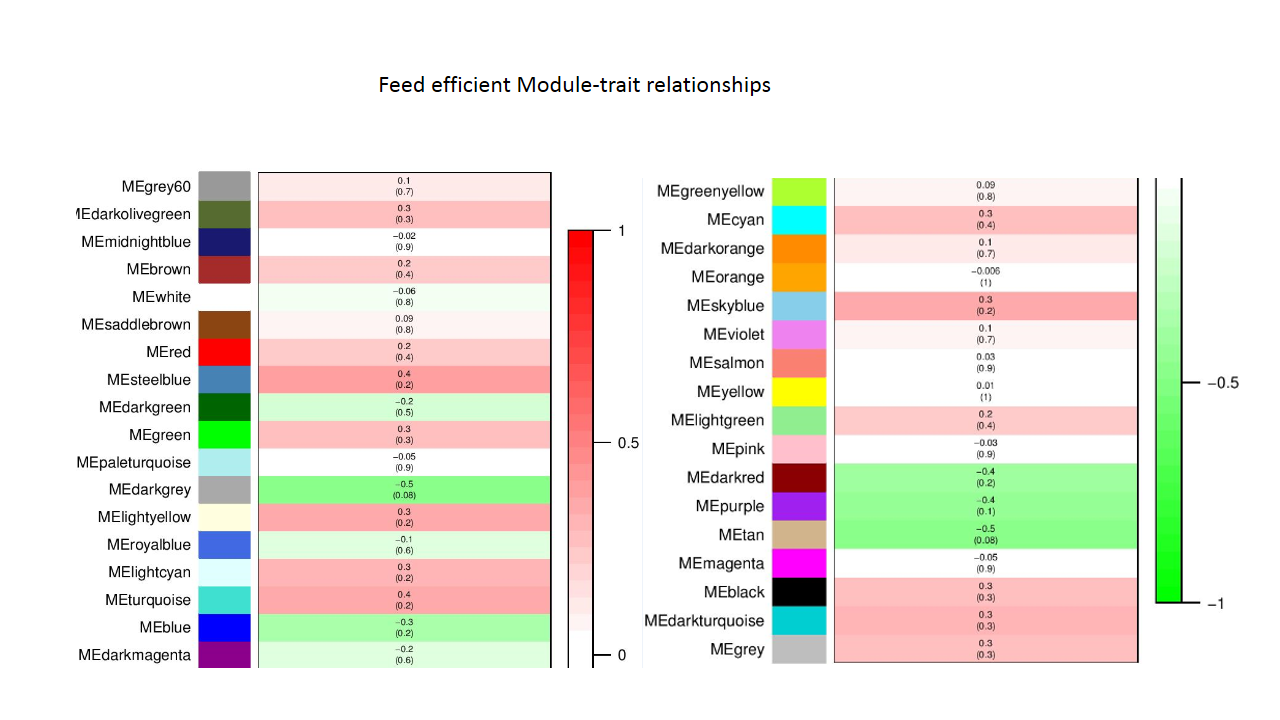


**Supplementary Figure S11.** Module-trait relationship from Feed efficient mRNA modules in skeletal muscle


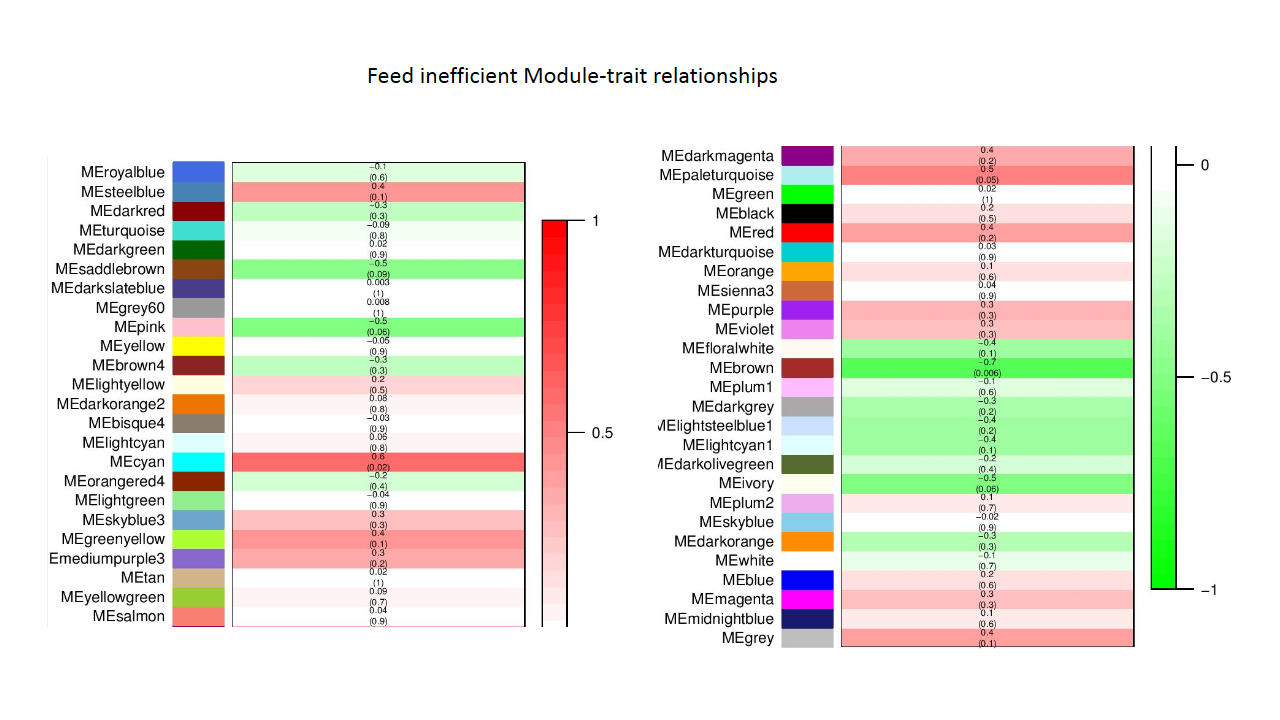


**Supplementary Figure S12.** Module-trait relationship from Feed inefficient mRNA modules in skeletal muscle.


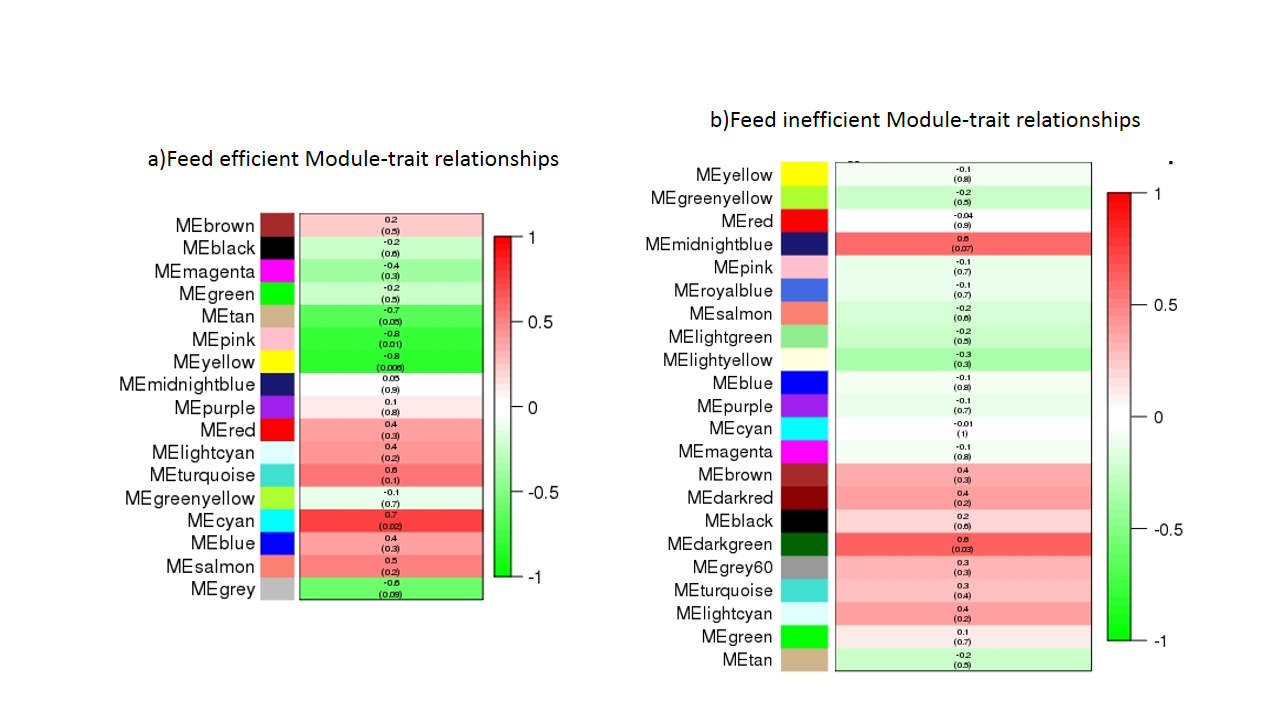


**Supplementary Figure S13.** Module-trait relationship from a) Feed efficient and b) Feed inefficien miRNA modules in liver.


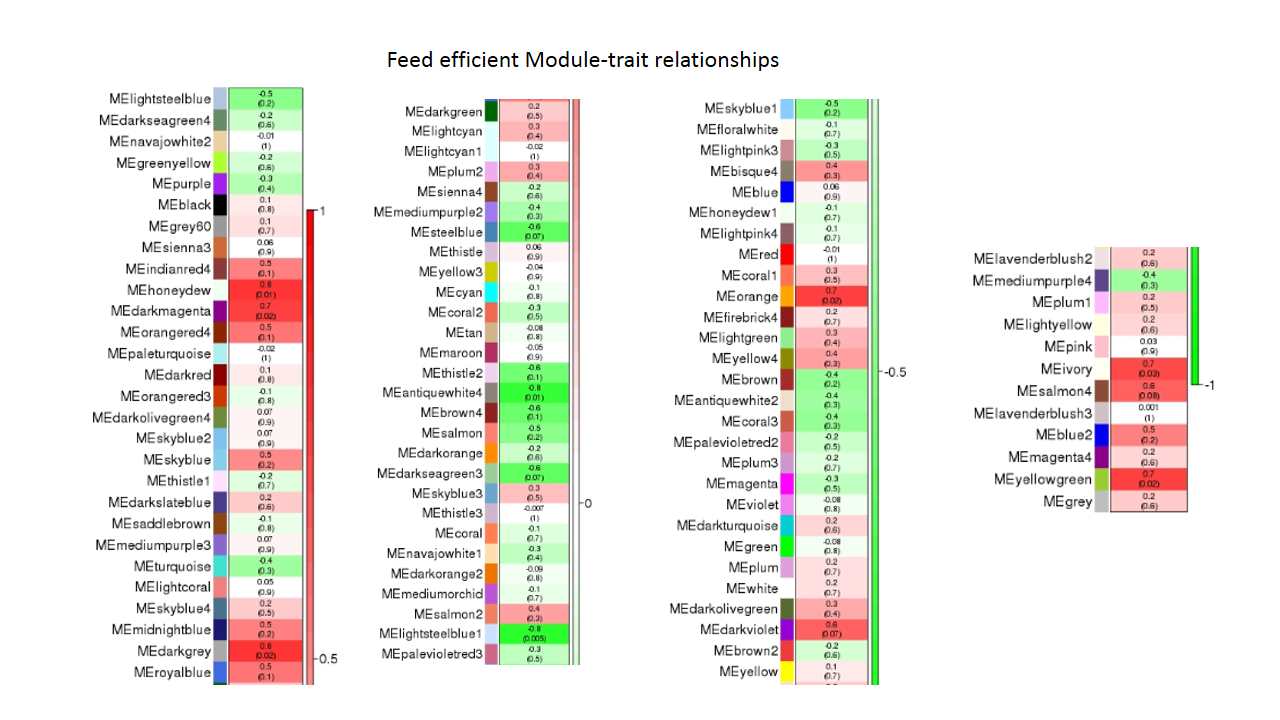


**Supplementary Figure S14.** Module-trait relationship from Feed efficient mRNA modules in liver.


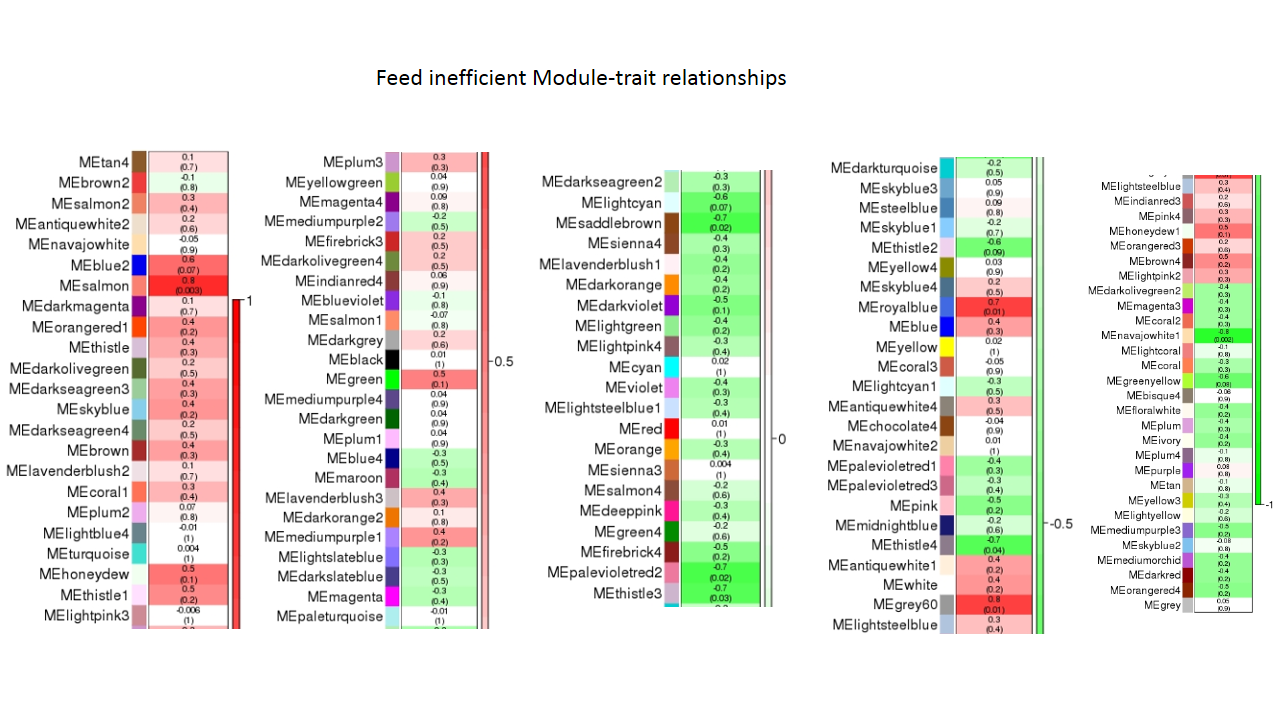


**Supplementary Figure S15.** Module-trait relationship from Feed inefficient mRNA modules in liver.


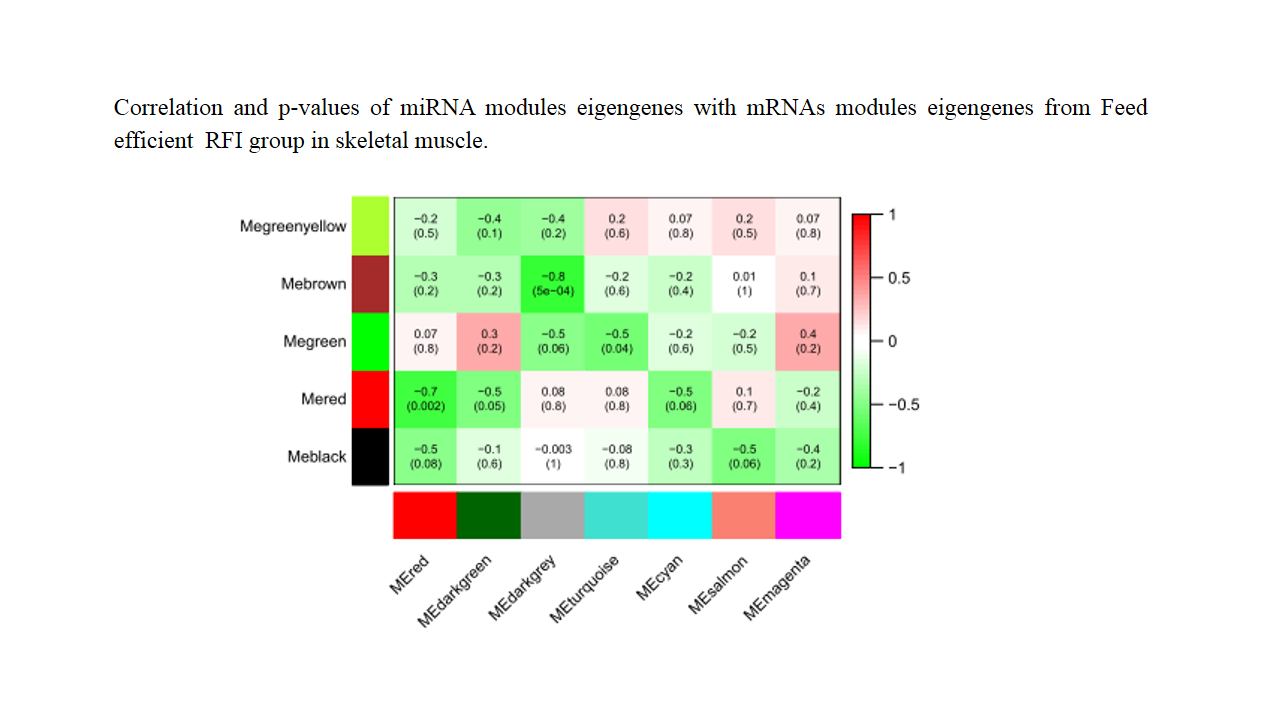


**Supplementary Figure S16.** Correlation and p-values of miRNA modules eigengenes with mRNAs modules eigengenes from Feed efficient RFI group in skeletal muscle.


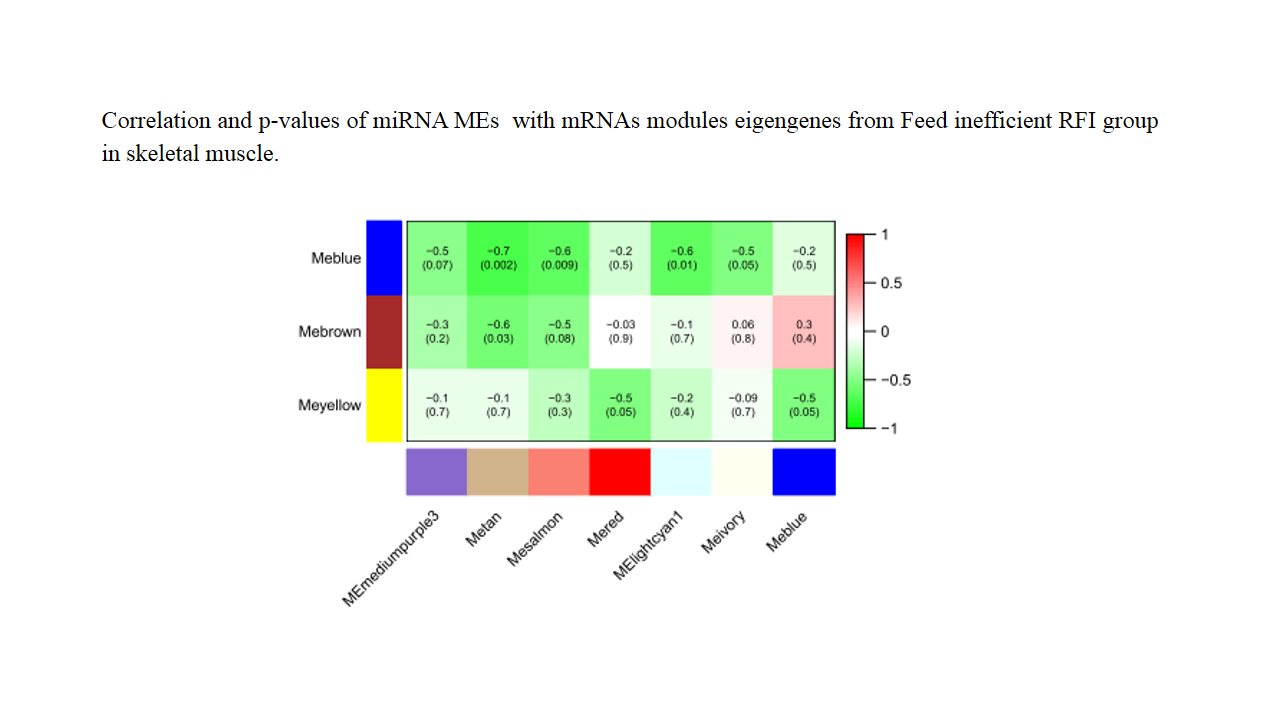
**Supplementary Figure S17.** Correlation and p-values of miRNA modules eigengenes with mRNAs modules eigengenes from Feed inefficient RFI group in skeletal muscle.


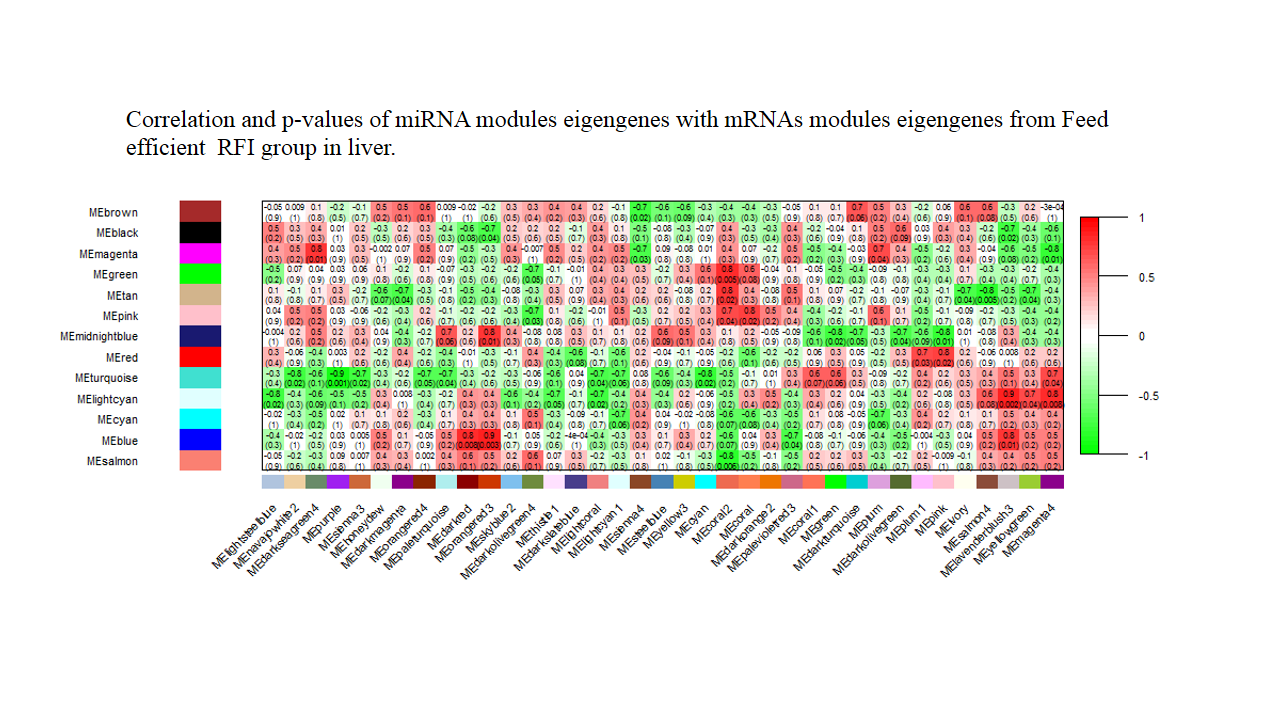
**Supplementary Figure S18.** Correlation and p-values of miRNA modules eigengenes with mRNAs modules eigengenes from Feed efficient RFI group in liver.


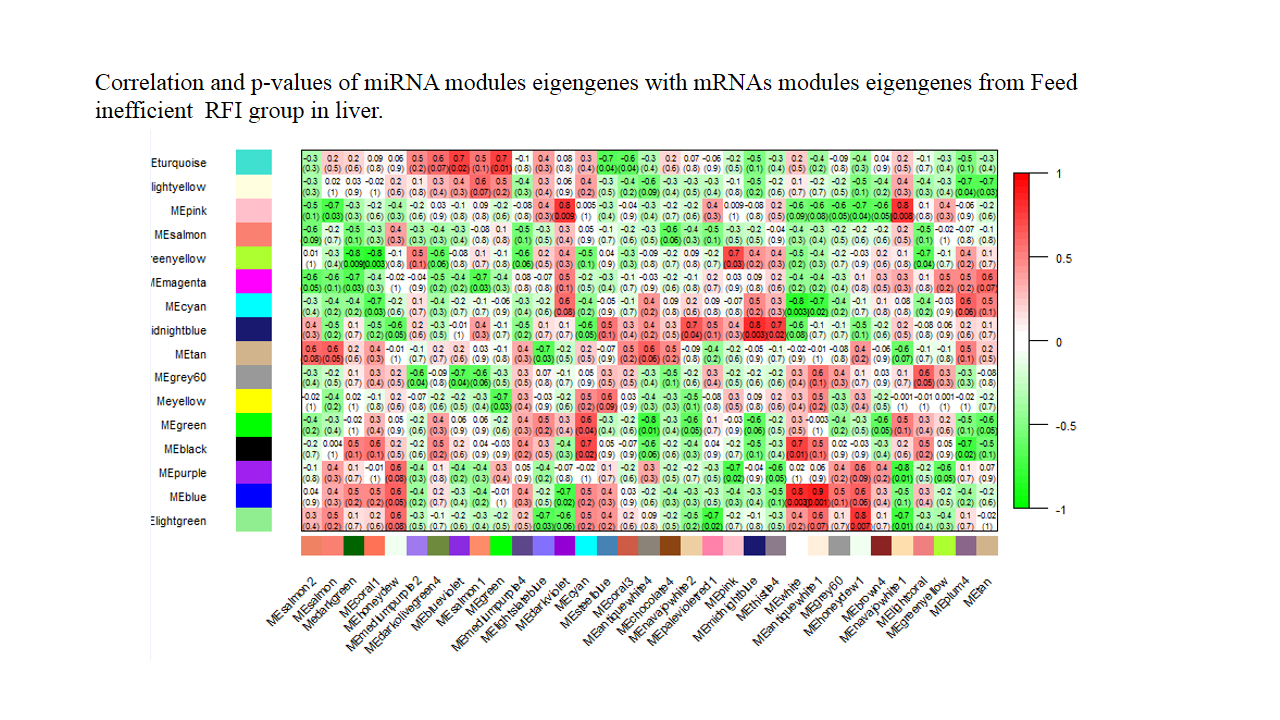


**Supplementary Figure S19.** Correlation and p-values of miRNA modules eigengenes with mRNAs modules eigengenes from Feed inefficient RFI group in liver.
